# Supplementary material for: Feasibility and usability of a very low-cost bubble continuous positive airway pressure device including oxygen blenders in a Ugandan level two newborn unit
Source: PLOS Glob Public Health. 2023 Mar 8;3(3):e0001354. doi: 10.1371/journal.pgph.0001354 (PMC10021653; doi:10.1371/journal.pgph.0001354)
Supplement: S1 File — (PDF) [file pgph.0001354.s002.pdf]

APPENDIX C.
BCPAP THERAPY GUIDELINES OF CARE

This guideline is designed to provide information to assist trained health professionals in decision making and is based on best evidence available at the time the guideline was developed. It should be used strictly as a guideline and not as a substitute for proper training, or for the proper assessment of the individual needs of each patient. Please contact us to ensure you are using the current version of this guideline. Adara Development and its associates will not be liable to any person in any way (including for negligence) for any loss or damage in any way connected with the use of this guideline.

INTRODUCTION

Bubble Continuous Positive Airway Pressure (bCPAP) is a simple means of providing respiratory support to spontaneously breathing newborns with respiratory failure. It is a system that provides gentle, constant pressure to keep the airway and lungs open - helping to decrease the work of breathing.

With newborns, bCPAP can be used to manage various respiratory conditions that lead to respiratory failure. Some of these conditions include: respiratory distress syndrome (RDS), apnoea, transient tachypnoea of newborn (TTN), pneumonia and sepsis.

To provide bCPAP, five key components are needed:

1. A source of continuous pressure
2. Inspiratory tube
3. Nasal cannula
4. Expiratory tube
5. Reservoir bottle

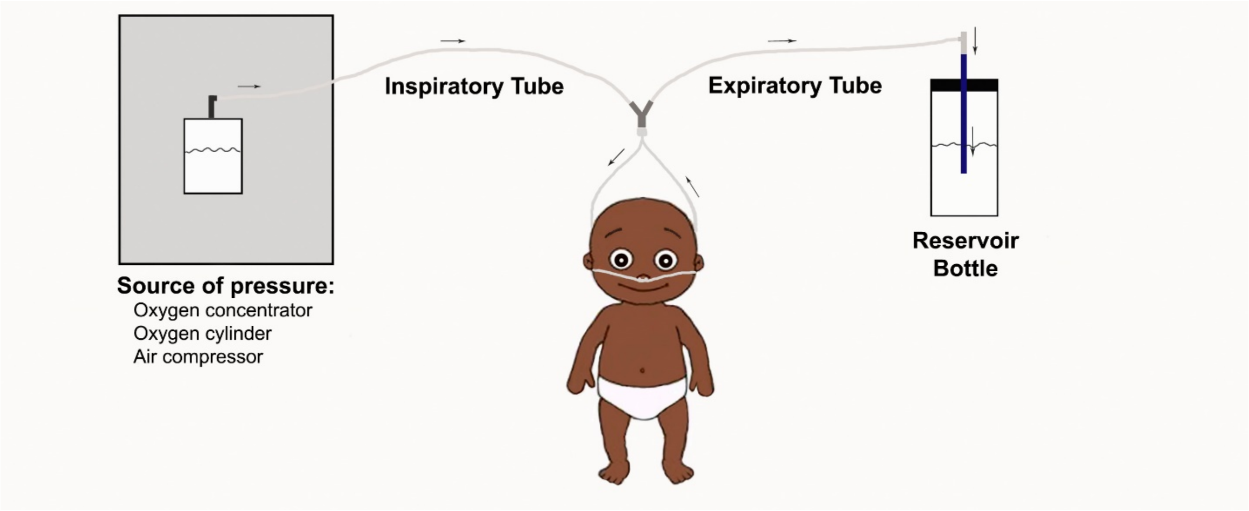

PROCEDURE

INITIATING BCPAP

	Procedure	Rationale
1.	Perform a complete **physical assessment** of the infant including:<br>• Respiration: rate, effort, breath sounds, signs of distress (tachypnoea, nasal flaring, sternal indrawing, rib retractions, grunting)<br>• Cardiovascular: auscultation, central and peripheral perfusion (capillary refill), blood pressure (if available)<br>• Neurological: muscle tone, response to stimulation and activity<br>• Gastro-intestinal: specific characteristics (e.g. cleft palate, omphalocoele), abdominal distension, visible loops, bowel sounds<br>• Thermoregulation: infant and environmental temperature<br>• Oxygen saturation<br>• Abdominal girth	• A baseline assessment is essential for discovering underlying conditions, detecting changes in status and in evaluating effectiveness of bCPAP.<br>• The flow of bCPAP can enter the oesophagus and into the stomach, and lead to abdominal distention. A baseline abdominal girth will help monitor for severity of distention.<br>• Starting treatment with bCPAP as soon as possible is important for better outcomes. If the infant has signs of significant respiratory failure, start treatment with bCPAP immediately (unless contraindicated). Complete a full assessment as soon as possible after starting bCPAP therapy. Document time of assessment on the patient chart.
2.	Assess the **Respiratory Severity Score (RSS)**. Document the RSS on the patient chart and discuss with the medical team if bCPAP therapy is indicated.<br>RSS of 5 or more may indicate the need to start bCPAP.	• There are some instances where bCPAP may be indicated with an RSS of less than 5, or not be indicated even though the RSS is more than 5.<br>• The RSS is meant to be a guide and therefore it is important to assess all clinical indicators before starting bCPAP.

RSS: RESPIRATORY SEVERITY SCORE

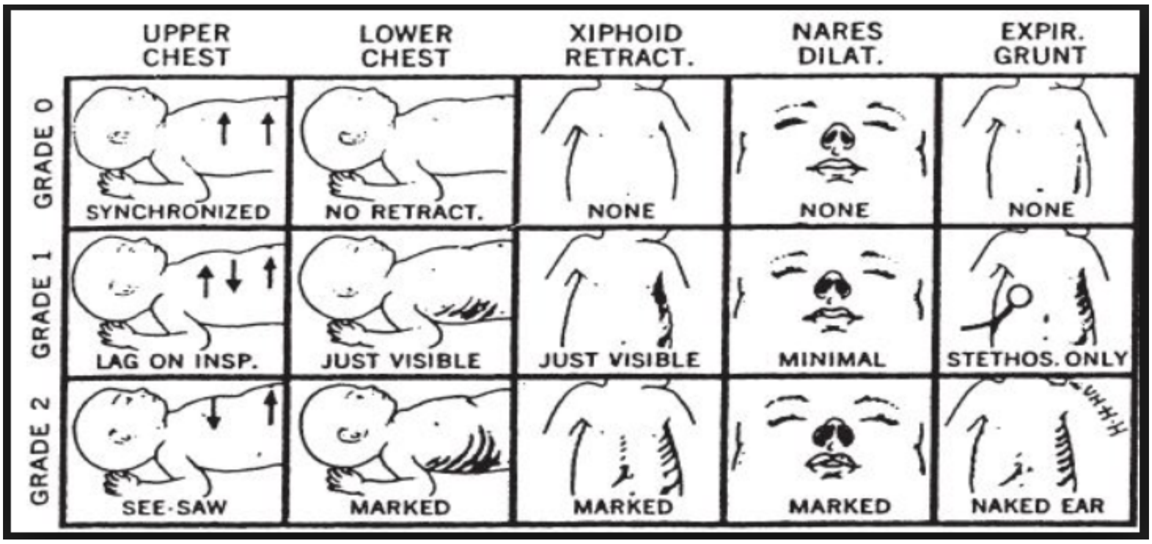

__________ + __________ + __________ + __________ + __________ **= RSS**

Respiratory Severity Score modified from: Silverman and Andersen, Pediatrics 1956; 17:1

	Procedure	Rationale
3.	Place the infant on a **pulse oximeter** to monitor oxygen saturations. Continuous oxygen saturation monitoring should be provided when initiating bCPAP and when the infant's condition is unstable.	• Oxygen saturations are a way to evaluate the effectiveness of bCPAP therapy and to detect changes in the infant's status. Oxygen saturations will help determine the oxygen requirements of the infant and indicate when adjustments should be made.
4.	If possible, **position the infant** with the head of the bed elevated to around 30°. If the infant is supine, place a small roll under the neck and shoulders. The roll should be firm enough to support the infant's head in the "sniffing" position.	• Elevating the head of the bed may decrease the pressure on the infant's diaphragm, making it easier to breathe.<br>• Only raise the head of the bed if it is safe to do so. Some beds are not designed to safely do this.<br>• Slight extension of the neck will keep the airway open.
5.	Using a bulb syringe, gently **suction** the mouth, nose and pharynx to ensure a clear airway.	• Secretions can block the prongs or the airway. This can worsen the symptoms of respiratory failure and cause apnoea or bradycardia.<br>• Gentle suctioning is required to prevent damage to the tissues. Tissue trauma can lead to an increase in secretions, further complicating treatment.
6.	Insert an **orogastric tube** and secure in place. Use the largest size that is appropriate for the patient (6-8fr). Remove the plunger from a 20ml syringe and connect it to the end of the OG tube. Leave the syringe open and raise the end of the syringe to prevent stomach contents from draining out the tube.	• The flow from the bCPAP system can enter the oesophagus and into the stomach, and lead to abdominal distention. The OG tube will help extra air leave the stomach.<br>• Small diameter tubes do not remove extra air from the stomach well.<br>• See NG/OG GOC for more information on placing OG tube.
7.	Select **proper size cannula** by using size guide. Ideal prongs size will fill approximately 80% of nares. Ensure that prongs do not fill nares completely.<br>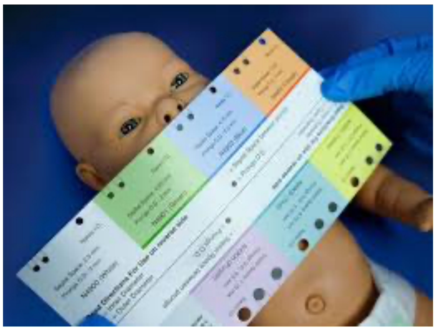	• Proper sizing is important to ensure that there is a good seal around the prongs and pressure is delivered to the lungs.<br>• Prongs that are too large place pressure on the nares and lead to tissue damage.<br>• Prongs that are too small may damage the nasal septum or cause a loss of pressure from air leaking around the prongs.
8.	**Set up bCPAP kit** per the Bubble CPAP Manual. Check the following for proper set-up:<br>• All connections are secure	• For safety, the bCPAP system should be completely set up, adjusted and checked before placing the system on the infant.

	Procedure	Rationale
	• Water level of reservoir bottle is at FILL LINE<br>• bCPAP system is connected to pressure source (oxygen and/or air)<br>• Water level in oxygen concentrator humidifier bottle is adequate<br>• Oxygen and/or air flow is set at 3 litres or more to ensure continuous bubbling in the reservoir bottle<br>• bCPAP pressure is set to the ordered amount	This will avoid inadvertently delivering sudden or high pressure to the infant during set-up.
9.	Place the **prongs on the infant** and ensure the following:<br>• Prongs are placed into the nares curved side down.<br>• There is a small space between the tip of the septum and the prongs<br>• Cannula are secured by sliding the ring towards the back of the infant's head. Ensure the infant does not lie directly on the ring.	• Correct positioning of the prongs reduces the risk of trauma and ensures effective delivery of bCPAP.<br>• The best way to avoid tissue damage is to leave a small space (or "cushion of air") between the septum and the prongs.<br>• The nasal septum of the infant is fragile. Breakdown of the skin can occur with pressure, friction or moisture. The site should be monitored frequently to ensure proper prong placement and to avoid skin breakdown.<br>• Lying on the ring can cause a pressure sore on the back of the head.
10.	Gently clean the infant's cheeks with water and dry thoroughly using a soft gauze. **Tape the prongs to the face** carefully on each side. Ensure they are flat, straight and not twisted.	• Cleaning the face helps the tape to stay in place.<br>• Prongs that are rotated and angled too far down can be occluded by the tissue of the nares. Twisting of the prongs can create pressure points in the nares and lead to tissue damage.
11.	Once applied, check the system:<br>• View the reservoir, checking that bubbles are continuously present. There should **not be any pauses in bubbling during the respiratory cycle**. Adjust the flow to achieve gentle, constant bubbling.	• Constant bubbling ensures that there is adequate flow to clear the CO2 in the system.<br>• If the system is not bubbling after checking the system, consult the bCPAP Manual for troubleshooting tips.
12.	• **Monitor** oxygen saturations and adjust amount of oxygen delivered as needed.<br>• **Repeat RSS** and record score on patient chart.	• Repeating the RSS helps to evaluate the effectiveness of the bCPAP therapy.

MAINTENANCE OF BCPAP

	Procedure	Rationale
1.	After placement on bCPAP, perform infant assessment and vital signs as follows:	• The infant on bCPAP will require some time to stabilize and should be closely monitored during this time.

	Procedure	Rationale
	• **Every 15-30 minutes in the first hour** after being placed on bCPAP<br>• **Every 3 hours after infant's condition stabilizes**<br>• Patient who remains unstable should be monitored more regularly as needed until stable	• Initially, adjustments may be necessary until the correct amount of bCPAP support is identified.<br>• The condition of a baby receiving bCPAP can change quickly. Frequent, close monitoring is required for the duration of bCPAP therapy.
2.	Provide **continuous oxygen saturation** monitoring to assess for infant's oxygen requirements.	• Too much or too little oxygen can be harmful for the infant. Adjustments must be made according to the oxygen saturations of the infant.
3.	**Ensure clear airways** through careful assessment and gentle suctioning when needed:<br>• When there is no heater/humidifier in use, apply 1-2 **drops of 0.9% saline solution** in each nostril every 3-4 hours at care times. This provides moisture to the airway and mucosa to prevent drying of the nasal passages and thickening of nasal secretions.<br>• Carefully **suction** the mouth, nose and pharynx when the infant shows signs of blocked airways. Gently suction using a bulb syringe. Saline drops will help loosen secretions that have hardened. This process may need to be repeated if you are unable to clear the airway on the first attempt. Repeat, allowing the baby to rest between suctioning, until secretions blocking the airway are cleared. See Suctioning Procedure for more information.<br>• If the bulb syringe does not clear the airways and a suction machine is available, attempt to remove hardened secretions with an appropriately sized catheter. The suction should not be set any higher than 80-100mmH2O.	• Oxygen/air delivery can dry the tissues of the nose and throat. Keeping the airways moist can prevent secretions from drying and blocking the airways.<br>• The presence of secretions will narrow or even block the airway and increase the effort of breathing. This can cause increased oxygen requirement, obstructive apnoea, bradycardia or pneumothorax.<br>• Infants breath through their noses. If their nasal passages are blocked, you will note severe respiratory distress and a decrease in oxygen saturations. The infant may puff their cheeks and attempt to breathe through their mouth.<br>• Gentle suctioning is required to prevent damage to the tissues. Tissue trauma can lead to an increase in secretions, further complicating treatment.<br>• Use of a catheter can damage the tissues of the nasal passage if too much force is used. Caution should be used when using a suction machine and catheter.
4.	**Change the infants position every 3-4 hours** with vital signs/assessments and document on patient chart. Ensure proper developmental positioning of the infant.<br>• When supine or side-lying, support the airway using a neck roll<br>• When positioned prone, remove the neck roll and use a chest pad under the infant.<br>• See Neurodevelopmentally Supportive Care GOC for more information.	• Regular position changes are important for prevention of skin breakdown and promote neurodevelopmental support.<br>• A clear well-supported airway will assist the infant's respiratory effort.<br>• Proper positioning can decrease oxygen demand and improve oxygenation.

	Procedure	Rationale
5.	**Check for correct placement of OG tube** every 3 hours and before feedings. It may be necessary to gently aspirate air for accumulated air before feeding the infant by OG tube.<br><br>Keep the syringe attached to the OG tube but remove the plunger to allow extra air to leave the stomach.	• The flow of pressure from the bCPAP can enter the stomach and lead to abdominal distention.<br>• The OG tube left open and connected to a syringe (without the plunger) to help accumulated air leave the stomach.
6.	**Assess the skin surrounding the prongs** at least every 3-4 hours with vital signs/assessments.<br><br>• Check for proper nasal cannula prong size and placement. Ensure there is a space between the prongs and nasal septum. Check for pressure points from improper prong positioning.<br>• Keep the area dry and clean<br>• Remove the prongs if necessary, to get a clear view of the nares and septum.	• Improper sizing or positioning of nasal cannula can lead to skin breakdown. Frequent monitoring can prevent serious damage.<br>• Keeping the area dry will decrease the risk of skin breakdown.<br>• In some instances, it may be difficult to view the area surrounding the prongs because of the tape used to secure the prongs. However, it is very important to assess for skin breakdown before it occurs.
7.	Evaluate the bCPAP system:<br><br>• **Check the entire system** from pressure source - to the baby - to the reservoir bottle, **every hour** to ensure that it is functioning correctly.<br>• Use the *bCPAP System Checklist* and document findings once a shift.	• The bCPAP delivery system must be constantly evaluated to ensure proper treatment.<br>• Refer to the bCPAP Manual for detailed information.

MAKING ADJUSTMENTS TO BCPAP

	Procedure	Rationale
1.	Initial bCPAP setting is **5cm H2O.**<br><br>The usual range of settings 5-8cm H2O.	• When initiating bCPAP, start with 5cm H2O and increase from there as needed.
2.	An increase in bCPAP pressure may be needed from the initial setting. **Increase level of support for signs of respiratory failure**:<br><br>• RSS above 5<br>• Low oxygen saturation despite increasing percentage of oxygen delivered<br>• Apnoea	• When initiating bCPAP, the infant may require adjustments to the settings until the correct amount of support is identified.
3.	**Increase CPAP level by 1 cm H2O pressure at a time.**<br><br>• Monitor for changes in RSS, respiratory rate and oxygen requirement after each change.<br><br>• If the infant needs more support, make increases gradually (every 15-30 minutes), if the infant's condition allows. This allows time	• It may take time for the infant to show a response to bCPAP adjustments. Not allowing adequate time to assess the infant after a change can result in bCPAP adjustments being made too quickly.<br>• The infant may have areas of alveoli collapse. Higher initial levels of bCPAP support may be necessary until the

	Procedure	Rationale
	for the infant to adjust after each pressure change.<br>• If the infant's condition requires a rapid increase in bCPAP level, consider attempting to wean the level when the infant's condition stabilizes.<br>***BCPAP levels above 8cm H2O should be used with caution. Discussions and evaluation should take place with attending doctor.***	collapsed alveoli are re-opened. Once alveoli are re-opened, the infant may need less support.<br>• It is important to only use the amount of support needed and no more. Using too much pressure can lead to lung overdistention and pneumothorax.

TITRATING OXYGEN CONCENTRATION

	Procedure	Rationale
1.	Oxygen saturations should be monitored with the use of a pulse oximeter. The reading the pulse oximeter provides is *called peripheral oxygen saturation* or *SpO2*. Consult the chart below for appropriate SpO2 target range.<br>Set **pulse oximeter alarms 2 above and 2 below the target range** for the patient.	• The pulse oximeter reading of SpO2 is closely correlated with the arterial oxygen saturation, making it a valuable method for measuring oxygen saturation.<br>• Target ranges will vary according to patient's size and gestational age.<br>• Alarms are set to notify the staff when the patient's SpO2 falls outside the recommended range - and a corrective action should be taken.

	SpO2 target range (%)	Monitor Alarm Setting (%)	
		LOW	HIGH
Less than 32 weeks gestation or <1250 grams	88 - 95	86	97
Equal or greater than 32 weeks gestation and >1250grams	90 - 95	88	97

For an infant in room air (no supplemental oxygen), the high monitor alarm can be set at 100%.

	Procedure	Rationale
2.	**Adjust oxygen delivery to maintain oxygen saturations in the target range.**<br>The concentration of oxygen can be titrated using the following methods:<br>• Oxygen concentrator + Air compressor<br>• Air compressor alone<br>• Use of oxygen concentrator that delivers less than 100%<br>(See bCPAP Manual for more details on how to blend oxygen)<br>Oxygen concentrators are analysed for percentage delivered every 2 weeks or more frequently as needed.	• Delivering oxygen levels that are too high for the infant can cause complications, including blindness, brain and lung damage.<br>• Using both an air compressor and an oxygen concentrator together is a way to titrate the amount of oxygen to meet the patient's specific needs.<br>• Using an air compressor alone will supply air pressure (21% oxygen) when the infant does not need supplemental oxygen.<br>• Oxygen concentrators are tested and labelled with the concentration they supply.
3.	**Guidelines for titrating oxygen delivery:**<br>**Whenever saturation falls below SpO2 target range:**	• When the saturations fall, it is important to assess for reasons that might be

	Procedure	Rationale
	• Assess infant for changes in their condition (colour, work of breathing, breath sounds, heart rate, etc.)<br>• Troubleshoot bCPAP system (check system from end to end, check nasal interface, assess for secretions, etc.)<br>• Check pulse oximeter sensor and reposition or replace as needed<br>• Reposition the infant if necessary (for better airway or comfort)<br><br>**When oxygen saturation falls below SpO2 target range (but still at or above 80%) for 5 minutes:**<br><br>**OR**<br><br>**When the oxygen saturation falls below 80% for 1 minute:**<br><br>• Increase percentage of oxygen delivered<br>• Monitor saturations closely for 10-15 minutes after making the change<br><br>***If the oxygen saturation drops severely and the baby needs emergency attention:***<br><br>• *increase oxygen percentage immediately.*<br><br>**When the oxygen saturation stays above target range for 5 minutes**<br><br>Decrease percentage of oxygen delivered	contributing to the situation. Start with assessment of the baby.<br>• See Bubble CPAP Manual for more information.<br><br>• An older pulse oximeter probe may have lost its' adhesive. Reposition, re-secure or replace probe as necessary.<br>• The infant's airway is soft and can be blocked if not positioned properly. Placing the baby prone can improve oxygenation.<br><br>• If after proper assessment, the oxygen saturation level remains low, the infant requires a higher level of oxygen to bring the oxygen saturations into the target range.<br><br>• After making a change in the oxygen concentration delivered, monitor the infant for desired response. Make additional adjustments as needed.<br><br>• Small, gradual changes in oxygen amount are better than large changes to avoid the saturations going from "too high" to "too low".<br><br>• In emergency situations, increase the oxygen concentration immediately to the amount necessary to resuscitate the infant (may require 100%).<br><br>• When making decreases to oxygen delivered, make small changes and evaluate infant's response before making additional changes.

WEANING OFF BCPAP SUPPORT

	Procedure	Rationale
1.	Over the course of treatment, as the patient recovers - decreases in bCPAP support can be made. Consider decreasing bCPAP level when:<br><br>• RSS is less than 4<br><br>• Oxygen saturations are consistently above 90% and the infant is receiving a low amount of supplemental oxygen<br><br>• There is no tachypnoea<br><br>• The patient has minimal to no apnoea or bradycardia events	• RSS of 4 indicates improving respiratory status<br>• If an infant is requiring a high amount of oxygen, it may indicate that the baby is not ready to decrease support.<br>• The absence of tachypnoea, apnoea and bradycardia is an indication of improving respiratory status
2.	Decrease bCPAP level by 1 cm H2O every 12-24 hours and monitor for:	• An increase in RSS, respiratory rate or oxygen requirement are signs that the

	Procedure	Rationale
	• Increase in RSS or respiratory rate<br>• Increase in oxygen requirement	infant is not tolerating the decrease in bCPAP support.

REMOVAL OF BCPAP

	Procedure	Rationale
1.	Patient is ready to trial off bCPAP when they are:<br>• Stable on bCPAP of 5cm H2O<br>• Oxygen saturations are consistently above 90% and the infant is receiving a low amount of supplemental oxygen<br>• RSS less than 4<br>• There is no tachypnoea<br>• The patient has minimal to no apnoea or bradycardia events	• Do not decrease bCPAP level below 4cm H20<br>• RSS of 4 indicates improving respiratory status<br>• If an infant is requiring a high amount of oxygen, it may indicate that the baby is not ready to decrease support.<br>• The absence of tachypnoea, apnoea and bradycardia is an indication of improving respiratory status.

BCPAP COMPLICATIONS AND MANAGEMENT

Complication	Management
**Mild complication:**	
Nasal irritation	• Provide diligent skin care<br>• Ensure proper fit, size, and position of cannula, add padding when indicated
Abdominal distention	• Ensure orogastric tube is allowing accumulated air in the stomach to be vented between feeds<br>• Position infant prone
**Moderate complication:**	
Nasal breakdown	• Provide diligent skin care<br>• Ensure proper fit, size, and position of cannula, add padding when indicated<br>• Consider short time periods off bCPAP if patient can tolerate it or discontinue bCPAP therapy if appropriate
Feeding intolerance	• Exam/review to screen for abdominal pathology<br>• Ensure orogastric tube is allowing accumulated air in the stomach to be vented between feeds<br>• Consider adjusting feeding volume or timing<br>• Position infant prone
**Serious complication:**	
Hypoxia/Hyperoxia	• Continuous monitoring with pulse oximetry<br>• Adjustment of oxygen concentration according to patient need
Pneumothorax	• Early identification (diagnosed by sudden deterioration, unilateral loss of breath sounds, bedside X-ray or ultrasound as available)<br>• Needle thoracentesis by trained medical personnel
