# Supplementary material for: Feasibility and usability of a very low-cost bubble continuous positive airway pressure device including oxygen blenders in a Ugandan level two newborn unit
Source: PLOS Glob Public Health. 2023 Mar 8;3(3):e0001354. doi: 10.1371/journal.pgph.0001354 (PMC10021653; doi:10.1371/journal.pgph.0001354)
Supplement: S2 File — (PDF) [file pgph.0001354.s003.pdf]

 (continued from page 3)

G. SPECIFICATIONS

- Storage temperature: -20°C to 50°C
- Operating environmental conditions: up to 30°C at 75% relative humidity
- Oxygen source: 20 psig at 10 LPM; limited operation can be possible with a lower pressure source
- FiO2 variation: ± 5% from nominal blend with 100% source of oxygen
- Delivered pressure range: 4 to 8 cmH2O with increments of 1 cmH2O
- Flow delivered to patient range: 2 to 5 LPM
- Patient interface: four sizes of nasal prongs
- Sound level: up to 80 dBA at 50 cm distance
- Bubbler water fill volume: 800 ml

CAUTIONS and WARNINGS

1. Blender oxygen ratio is based on a 100% source of oxygen; lower source concentrations yield lower blends.
2. Keep blender free from obstruction and at least 50 cm away from infant to minimize harmful sound levels.
3. A blender installed in the reverse direction will not work correctly. Flow and pressures will be much lower for the same oxygen input, and FiO2 can potentially be higher than labeled.
4. Flow rates higher than indicated in Table 2 (see section F) can produce elevated FiO2 levels in the blenders.
5. Ensure bubbler vent is free from obstruction. Occlusion can cause the patient to receive high levels of pressure.
6. Using this bCPAP device in dry environments or at high altitude can cause excessive drying of nasal passages. Extra monitoring and intervention may be required in these environments.
7. Do not replace any components with non-original or reprocessed parts.
8. Kit should be used only with appropriate auxiliary equipment, including orogastric tube, pulse oximetry and a suction device.
9. Kit should be used only by trained providers familiar with administering bCPAP therapy with blended air-oxygen.
10. Device is single use: cleaning can negatively impact performance.
11. Properly dispose of kits to prevent reuse or repurposing.
12. Store in box until use.
13. This is an investigational device, limited to investigational use only.

SINAPI biomedical

ARC Infruitec North Campus,
Lelie Road, Stellenbosch, 7600, South Africa
Tel: +27 21 887 5260 Fax: +27 21 887 3059
email: sales@sinapibiomedical.com

REF **bCPAP**

SINAPI biomedical

CONTENTS: 1 x bubble CPAP (continuous positive airway pressure kit with oxygen blenders

SINAPI

bubble CPAP kit

To provide respiratory support to a neonate in respiratory distress and where other means of respiratory support are not available

CAUTION: Federal law restricts this device to sale by or on the order of a physician.

Do not use if unit package is opened or damaged.

NON STERILE

Single Use

If re-used the sterility of the device is compromised, which will greatly increase infection risk

See Instructions for Use

0°C 50°C Avoid Extreme Temperatures

LATEX Latex free

PHT **DEHP**

SINAPI biomedical, ARC Infruitec North Campus
Lelie Road, Stellenbosch, 7600, South Africa
Tel: +27 21 887 5260 Fax: +27 21 887 3059
email: sales@sinapibiomedical.com

LOT

Use By

MANUFACTURED IN SOUTH AFRICA

EC REP mdi Europa GmbH, Langenhagener Str. 71, D-30855 Hannover-Langenhagen

INSTRUCTIONS FOR USE : Page 2

INSTRUCTIONS FOR USE

A. DESCRIPTION

The bubble continuous positive airway pressure (bCPAP) kit with oxygen blender is a single-patient, disposable device intended to provide respiratory support to spontaneously breathing neonates with respiratory distress syndrome. The device is intended for use in health care facilities with access to compressed oxygen (at least 5 LPM). The device is pre-assembled and ready to use at the point of care.

B. INDICATIONS

The SINAPI bCPAP kit is intended to:

1. Provide required continuous distending pressure to spontaneously breathing preterm and full-term neonates up to 4 kg -
   a) In respiratory distress with atelectasis/prematurity (surfactant deficiency).
   b) Where other means of respiratory support are not available.

C. CONTRAINDICATIONS

- Patients with irregular, intermittent, or not spontaneous breathing patterns
- Patients over 4kg in weight.
- Extended treatment greater than 7 days.

D. SETUP

1. Remove the product from the package.
2. Flow delivered to the patient depends on the size of the patient's nares, source flow and blender used. Adjust source flow to produce bubbling (Table 1).
3. Cut and apply tape to the patient's cheeks to protect the skin beneath the tubing.
4. Insert and secure an orogastric tube.
5. Place bubbler bottle in bubbler stand.
6. Add 2.5 ml of acetic acid (vinegar) to the bubbler bottle and fill to the fill-line with distilled water.
7. Attach bubbler lid and insert straw.
8. Finish assembly of bCPAP circuit according to Figure 1, using the 37% blender.

SIZE	APPROXIMATE WEIGHT
Micro Preemie	<750 g
Preemie	750 - 1000 g
Newborn	1000 - 2500 g
Infant	>2500 g

Table 1: Cannula sizing chart

E. APPLICATION OF THE DEVICE

1. Attach the bCPAP circuit to the oxygen source.
2. Attach the pulse oximeter probe to patient and take a reading.
3. Before applying the cannula, set source oxygen flow to achieve the desired blended flow based on Table 2 (see section F). This will typically be from 3 to 5 LPM of oxygen flow, but it can be greater for larger neonates.
4. Move the bubbler straw up or down to the desired depth (typically 4 to 8 cm) based on patient indications. If bubbler begins to bubble with no resistance on the prongs, do not administer care, and proceed to troubleshooting. Block the cannula to check that this produces bubbles.
5. Insert the cannula and ensure a good fit. Use a foam seal around the prongs if helpful.
6. Securely attach cannula to patient's face using tape. Slide the silicone ring on the cannula snug to the patient's head.

F. MAINTENANCE OF THERAPY

1. Continuously monitor the patient and check the circuit hourly to ensure proper operation. Check should include:
- Flow is at the desired level and producing a steady stream of bubbles.
- The water level is full.

2. Every 3 to 4 hours:
- Apply saline drops to nose to prevent drying.
- Check for buildup of secretions; suction if necessary.

O_2 SOURCE FLOW (LPM)	37% BLENDED FLOW (LPM)	60% BLENDED FLOW (LPM)
2	0 to 1.5	2.0 to 3.0
3	0 to 4.0	3.5 to 4.5
4	2.5 to 6.5	5.0 to 5.5
5	5.0 to 8.5	7.0
6	7.5 to 10.5	8.0
7	9.0 to 12.0	9.0

Table 2: Normal blended flow rate range

G. TROUBLESHOOTING

NO BUBBLING:
- Ensure oxygen source is providing adequate flow. If flow is maxed on a 5 LPM concentrator, a 10 LPM concentrator or cylinder may be required.
- Check tubing circuit for any abnormalities in setup, and make sure all connections are secure.
- Check fit of nasal cannula.

INTERMITTENT BUBBLING:
- Ensure oxygen source is providing adequate flow. If flow is maxed on a 5 LPM concentrator, a 10 LPM concentrator or cylinder may be required.
- Check tubing circuit for any abnormalities in setup, and make sure all connections are secure.
- If no problems are detected, increase oxygen flow until steady bubbling is achieved.

H. CONTENTS

1. Bubbler stand
2. Cannula adapter
3. Air-Oxygen blenders (37 and 60%)
4. Nasal cannula
5. Bubbler straw holder
6. Bubbler straw
7. Bubbler bottle (a) with lid (b)
8. Circuit tubing:
   (a) Expiratory limb (140 cm)
   (b) Inspiratory limb (50 cm)
   (c) Supply line (130 cm)

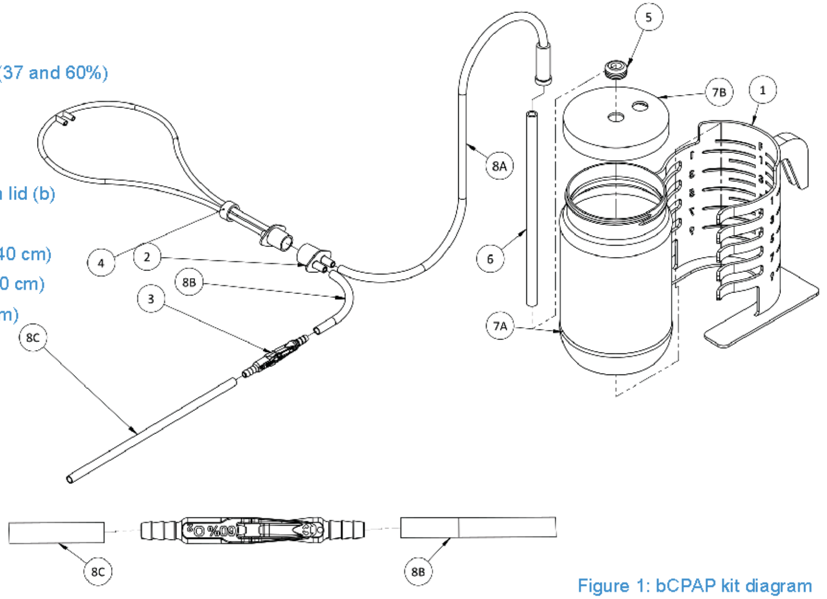

Figure 1: bCPAP kit diagram
