# Supplementary material for: Feasibility and usability of a very low-cost bubble continuous positive airway pressure device including oxygen blenders in a Ugandan level two newborn unit
Source: PLOS Glob Public Health. 2023 Mar 8;3(3):e0001354. doi: 10.1371/journal.pgph.0001354 (PMC10021653; doi:10.1371/journal.pgph.0001354)
Supplement: S3 File — (PDF) [file pgph.0001354.s004.pdf]

# Healthcare Worker Initial Interview Guide

**Date:** _______________________

**Data collector name:** __________________________________

**Instructions:** Up to 15 healthcare workers who were observed using the PATH bCPAP kit with blenders will be interviewed within the first five days of their first use of the PATH bCPAP kit with blenders.

**Interviewer name:** ____________________________________

1. HCW Study Number: (HCW ________ )
2. HCW job title (circle one): Nurse Midwife Doctor
3. Number of years of experience in the newborn unit: __________________
4. Prior to this study, have you used the Kiwoko Hospital bCPAP kit with air compressor before? How frequently?
5. Were you able to provide blended air and oxygen to all babies who needed it with the air compressor? What was your experience? (advantages, disadvantages)
6. How many times have you set up the PATH bCPAP kit with blender on an infant? _________________
7. What did you like the best about using the PATH bCPAP kit with blender? (if nothing mentioned, probe on the use of the blender)
8. What did you like the least about using the PATH bCPAP kit with blender? (if nothing mentioned, probe on the use of the blender)
9. How easy was it to use the PATH bCPAP kit with blender? Please explain the reasons for your answer.
10. What did you find difficult about using the PATH bCPAP with blender?
11. If you did not have pink tape or other special tape to secure the ram cannula, would you be able to use the kit? What would be the challenges of the kit if you didn't have this item?
12. If you did not have access to the foam pads, would you be able to use the kit? What would be the challenges of the kit if you didn't have this item?
13. How helpful is the nasal sizing guide? Do you think it should be part of the kit?
14. Did the two fixed rate blenders (37% and 60% oxygen) allow you to adequately blend oxygen to meet the patient's needs? Did you find you needed more blends? If so, what would be the ideal blends to have available?

15. What do you like/dislike about the way the PATH bCPAP kit with blender is packaged? How would you change the packaging to make it easier to use?

16. What would you change in the PATH bCPAP kit with blender?

17. After this study is over, would you like to continue using the PATH bCPAP kit with blender?

18. Do you want to add any comparative thoughts between the Kiwoko bCPAP kit with air compressor (KH existing system) with the PATH bCPAP kit with blenders as a practitioner (set up, time taken, ease of device set-up, learning the device, difficulty of use, satisfaction with device, other factors)?
