# Supplementary material for: Feasibility and usability of a very low-cost bubble continuous positive airway pressure device including oxygen blenders in a Ugandan level two newborn unit
Source: PLOS Glob Public Health. 2023 Mar 8;3(3):e0001354. doi: 10.1371/journal.pgph.0001354 (PMC10021653; doi:10.1371/journal.pgph.0001354)
Supplement: S4 File — (PDF) [file pgph.0001354.s005.pdf]

Supplement D.

Interviewer Positionality

HN who conducted the primary analysis identifies as a female registered nurse with a Bachelor of Science in Nursing (BSN). HN has 17 years of experience, providing visitational clinical support at the newborn care unit at Kiwoko Hospital and 33 years working in a level 4 NICU in the United States. Her role and relationship with hospital staff has been in an advisory clinical capacity. AC, the peer researcher, as referenced below, identifies as a female registered nurse with a BSN, MPH. She has no previous in-person experience at Kiwoko hospital.

Content Analysis Framework

HN completed two initial data readings of all 15 transcripts. After the initial data readings, HN began descriptive coding of all 15 transcripts. HN noted emerging themes in her initial data readings. To assess the credibility and dependability within our initial data analysis, three transcripts were sent to a peer researcher, AC. AC read through three of the interview transcripts and used a descriptive coding method. Upon completion of the coding process, both research team members met to discuss their findings, however, HN did not send her findings to AC until after their discussion to avoid biasing AC's results.

After dialogic engagement between the two research team members, a few themes were initially excluded as AC did not confirm them in her coding process as prominent themes. To assess the disputed themes between the two researchers, a key informant provided secondary analysis. After discussion, the key informant deemed the disputed codes as significant to the analysis despite disagreement from AC. Final count of the emerging themes related to usability and feasibility of the bCPAP kit are noted in the results section.
