# Supplementary material for: Feasibility and usability of a very low-cost bubble continuous positive airway pressure device including oxygen blenders in a Ugandan level two newborn unit
Source: PLOS Glob Public Health. 2023 Mar 8;3(3):e0001354. doi: 10.1371/journal.pgph.0001354 (PMC10021653; doi:10.1371/journal.pgph.0001354)
Supplement: S5 File — (PDF) [file pgph.0001354.s006.pdf]

FEASIBILITY OF USE OF THE PATH BCPAP KIT INCLUDING OXYGEN BLENDERS IN A NEONATAL POPULATION IN UGANDA

Principal Investigators:
James Nyonyintono, MBChB, M.Med. Surgery (Mak)
Anna Hedstrom, MD

Version Number: 1.5

Date:
14 April 2021

Makerere University School of Public Health
Higher Degrees, Research and Ethics Committee
Valid Thru:
04 MAY 2022

Table of Contents

Table of Contents 2
Research Team 3
Acronyms and Abbreviations 5
Operational definitions 5
Abstract 6
1.0 **CHAPTER ONE**: Introduction and Background 7
2.0 **CHAPTER TWO**: Literature review 10
3.0 **CHAPTER THREE**: Statement of the problem, Justification and Conceptual framework 12
- 3.1 Problem statement 12
- 3.2 Justification of the problem 12
- 3.3 Study Product 14

4.0 **CHAPTER FOUR**: Research questions, hypothesis and objectives 19
- 4.1 Hypothesis 19
- 4.2 General objective 19
- 4.3 Specific objectives 19

5.0 **CHAPTER FIVE:** Methodology 20
- 5.1 Study design 20
- 5.2 Study site 20
- 5.3 Sample size 21
- 5.4 Study population 23
- 5.5 Study procedure 24
- 5.6 Study variables
- 5.7 Data collection
- 5.8 Data management and analysis 32
- 5.9 Ethical considerations 34

References 42

Appendices

A. PATH bCPAP and Blenders Instructions for Use
B. Verification test report and Substantial Equivalence Table
C. bCPAP Therapy Guidelines of Care
D. Data collection forms
E. Consent form(s) and Standard Operating Procedure for Obtaining Consent

Makerere University School of Public Health • Higher Degrees, Research and Ethics Committee •
Valid Thru:
04 MAY 2022

Research Team

Role	Name, Title	Responsibilities
US Principal Investigator	**Anna Hedstrom, MD**<br>Assistant Professor/ Neonatologist, University of Washington	• Training<br>• Oversight - Overall study completion
Country Principal Investigator	**James Nyonyintono, MBChB, M.Med. Surgery (Mak)**<br>Clinical Programs Manager, Kiwoko Hospital	• Oversight - Day-to-day study oversight, ensuring that the study is conducted according to the protocol<br>• Reporting - Adverse event confirmation and documentation<br>• Protecting participants' rights<br>• Communicating questions, concerns, and/or observations to the principal investigator and/or coordinating centre
Sponsor - PATH	**Manjari Quintanar Solares, MD**<br>PATH Study Representative<br><br>**Patricia S. Coffey, PhD., MPH**<br>PATH Responsible Project Manager	• Providing and managing funding and resources for study implementation
Senior Advisor	**Maneesh Batra, MD, MPH**<br>University of Washington Professor/ Neonatologist	• Training<br>• Study Advisor
Newborn Unit Oversight	**Josephine Nakakande, RN**<br>Newborn Unit nurse-in-charge, Kiwoko Hospital	• Oversight - Guideline adherence for bCPAP usage<br>• Obtaining informed consent and enrolling participants<br>• Training - Guidance of training<br>• Oversight - Nurse oversight
Data Collection Oversight and Consent	**Beatrice Niyonshaba, MPH**<br>Research, Monitoring and Evaluation Officer, Adara Development (Uganda)	• Oversight of data recorders<br>• Obtaining informed consent and enrolling participants<br>• Reporting - Preparing and sending required reports to medical monitor
Data Recorders	Nursing students - Kiwoko Hospital School of Nursing	• Documentation - Via shift work, a data recorder will be in unit around the clock to record data on bCPAP patients

Makerere University School of Public Health • Higher Degrees, Research and Ethics Committee • Valid Thru: 04 MAY 2022

		• Documentation - Initial reports of adverse events
Data Entry team	Newborn unit data entry team (this team has experience with medical record data entry into online database)	• Documentation - Record data from forms into REDCap
Independent medical monitor	Dr. Flavia Namiiro<br>Ugandan paediatrician experienced in the care of neonates and use of bCPAP kits	• Monitoring moderate and severe adverse events
Makerere University Senior Advisor	**Peter Waiswa, MBChB, MPH, PhD**<br>Associate Professor, Makerere University School of Public Health,	• Oversight - Study Design Advisor
Study support	**Heidi Nakamura, RN BSN**<br>Global Health Director, Adara Development (USA)	• Oversight - Study logistic support<br>• Ensuring that all study staff meet regulatory and fulfill training requirements<br>• Training
Study support	**Madeline Vaughan, MIPH**<br>Senior Programmes Director, Adara Development (Australia)	• Oversight - Study logistic support
Device support	**Sierra Jessup, BS**<br>Product Development Technical Coordinator, PATH	• Oversight - Device support
Biostatistician	**Paul Mubiri, M.Stat**<br>Research Officer under PERFORM2Scale Project, Makerere University	• Reporting – Data analysis<br>• Documentation - Creation and management of Redcap database
Qualitative analysis Support	**Mohan Paudel, PhD, MPH**<br>Monitoring and Evaluation Manager, Adara Development (Australia)	• Oversight - Qualitative Analysis<br>• Reporting - Data analysis

Makerere University School of Public Health • Higher Degrees, Research and Ethics Committee •
Valid Thru:
04 MAY 2022

Acronyms and Abbreviations

CPAP = Continuous Positive Airway Pressure
bCPAP = Bubble Continuous Positive Airway Pressure
EFS = Early feasibility study
HCW= Healthcare worker
RSS = Silverman Anderson Respiratory Severity Score[1]
WHO = World Health Organization

Operational definitions

PATH bCPAP kit without blenders	bCPAP (tubing/ cannula/ bubbler) without blenders
PATH bCPAP kit with oxygen blenders	bCPAP (tubing/ cannula/ bubbler) with blenders
PATH blenders	only the PATH blenders

Kiwoko bCPAP refers to the pre-existing bCPAP that has been in place at Kiwoko hospital since 2012. It is assembled from spare parts and donated nasal cannulas.(12).

Makerere University School of Public Health • Higher Degrees, Research and Ethics Committee • Valid Thru: 04 MAY 2022

Abstract

Preterm birth is responsible for 1/3 of deaths under age five- the majority which occur in resource-constrained settings. Many of these deaths are due to respiratory failure which can be treated with bubble continuous positive airway pressure (bCPAP). Commercialized bCPAP devices are expensive, leading resource-constrained settings to make and use improvised devices. These improvised devices have not been tested for performance and run on 100% oxygen. WHO guidelines strongly advise against the use of 100% oxygen—particularly with preterm newborns where it can cause blindness, lung and brain injury.

To address this need, PATH has developed a low-cost bCPAP kit which includes oxygen blenders that do not require electricity nor a source of pressurized air to blend oxygen with air. We seek to conduct an early feasibility study to:

1. assess the operational feasibility of using the PATH bCPAP kit including, when appropriate, in-line oxygen blending on neonatal patients and
2. assess the usability and acceptability of the PATH bCPAP kit with oxygen blenders by healthcare workers.

Newborns will be treated with the PATH bCPAP kit and a subset of these will also be treated with the PATH blender as needed to provide blended oxygen. Results from this study will be used to identify appropriate modification to the use procedures and/or the device as needed. Once testing is completed and product revision finalized, the PATH kit and blenders will allow resource-constrained settings to provide rigorously tested bCPAP therapy and blended oxygen to patients with reduced risk of morbidity from oxygen toxicity.

Makerere University School of Public Health • Higher Degrees, Research and Ethics Committee • Valid Thru: 04 MAY 2022

1.0 CHAPTER ONE: Introduction and Background

Preterm birth is responsible for 1/3 of paediatric deaths under age five.[2] Infants born very early suffer from respiratory distress syndrome due to under developed lungs and this is a leading cause of their deaths.[3] An additional 15% of children under age five die from trouble breathing due to respiratory tract infections such as pneumonia.[2] Continuous positive airway pressure (CPAP) provides constant distending pressure to the lungs and decreases the work of breathing for an infant. It is the recommended standard of care for respiratory failure caused by respiratory distress syndrome or pneumonia, and should be started as soon as the diagnosis is made.[4,5] The pressure provided by bubble CPAP (bCPAP) is regulated by the depth of the tube in the water bottle. The gas flow through the tube creates bubbling, and the resulting oscillations are helpful to a patient's lungs. Because of this, bCPAP is considered superior to standard CPAP in treating neonatal respiratory failure. Commercialized devices are available (Figure 1)[6], but are rarely found in resource constrained settings due to the prohibitive cost.

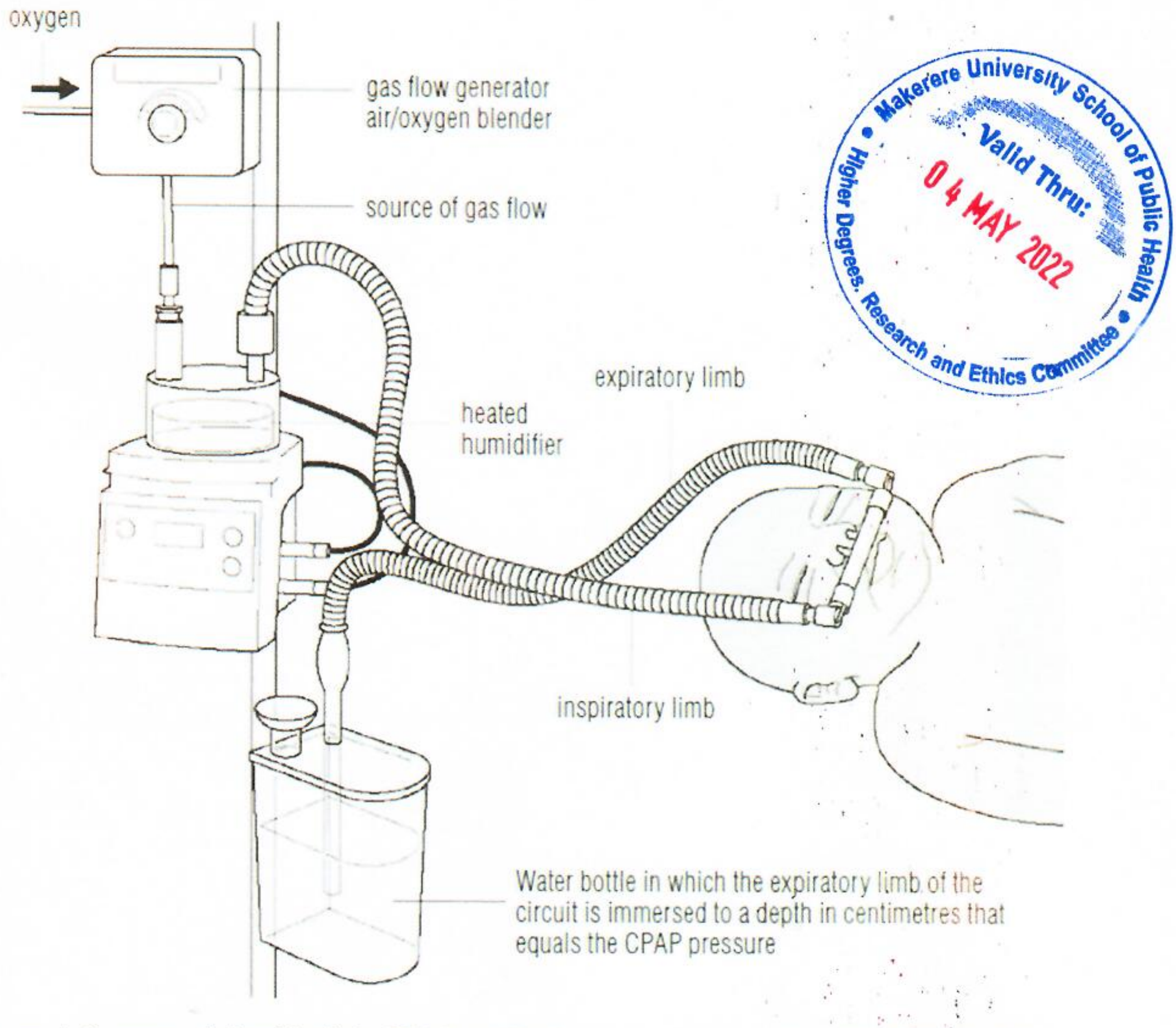

Makerere University School of Public Health • Higher Degrees, Research and Ethics Committee • Valid Thru: 04 MAY 2022

Figure 1. Commercialized bubble CPAP (bCPAP) circuit. These systems can cost up to 10,000USD. Figure from: [7]

Commercialized devices considered to be "low cost" do exist, however, at a price of US$900, resource-constrained sites still cannot afford them or cannot afford enough devices for the number of patients requiring treatment.[8] To address this care gap, the World Health Organization (WHO) recommends creating improvised bubble CPAP (bCPAP) devices using components available in the hospital, specifically tubing connected to bottles, with water to generate the necessary pressure.[7] (Figure 2). These improvised kits, however, have variable performance in terms of pressure delivered due to the variation in the diameter and length of available tubing, lack of appropriate cannulae sizes, and incorrect assembly of parts.

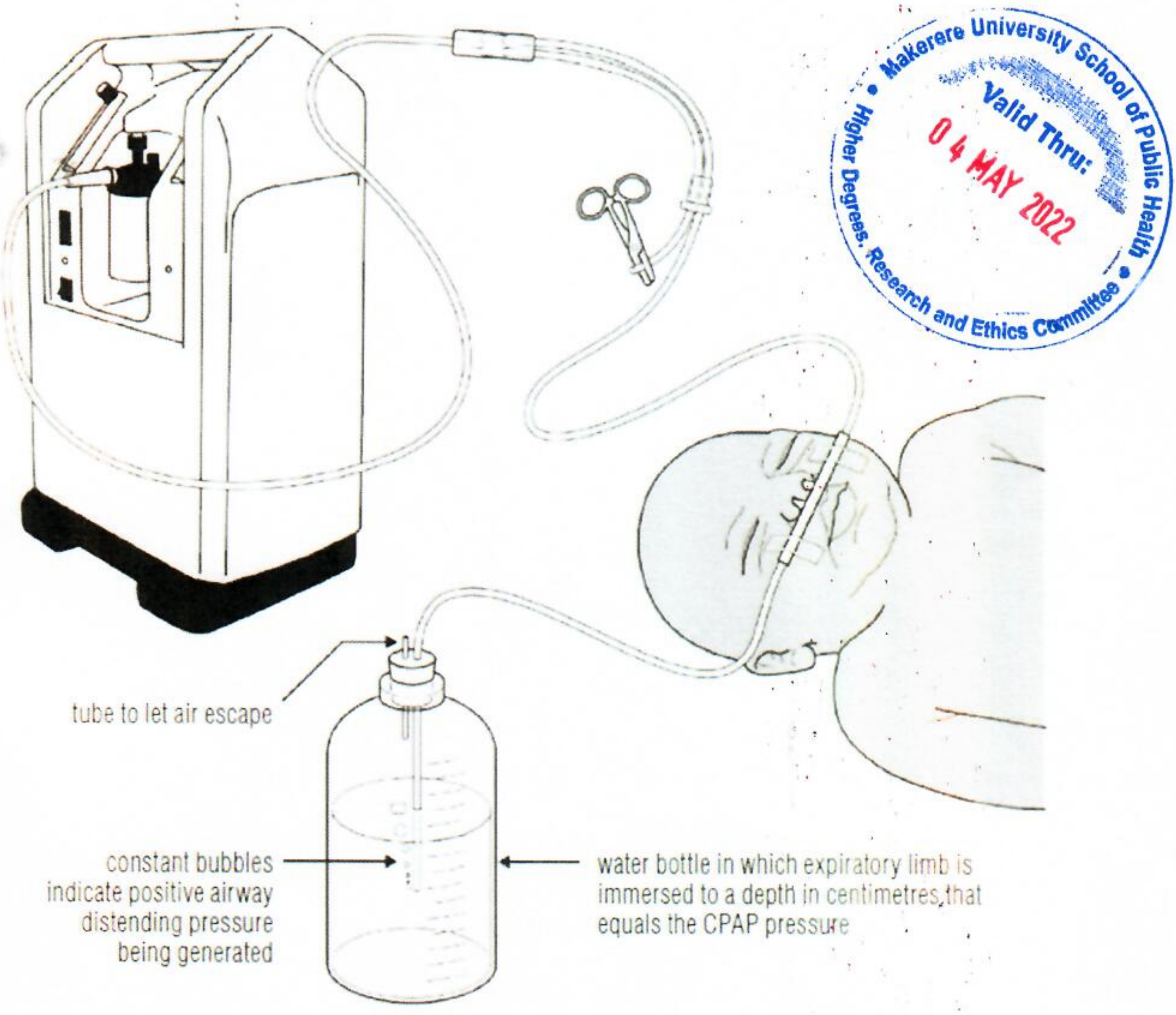

Makerere University School of Public Health • Higher Degrees, Research and Ethics Committee •
Valid Thru:
04 MAY 2022

Figure 2. Improvised bCPAP created from a simple nasal cannula, as advised by the WHO. Although lifesaving, this system runs on 100% oxygen which can be toxic to patients. Figure from [7].

Additionally, most resource-constrained settings providing bCPAP therapy do not have a mechanism to provide blended gases (air and oxygen) to the baby. WHO guidelines strongly advise against the use of 100% oxygen with newborns—particularly preterm newborns—as it poses a risk for blindness, chronic lung disease and brain injury.[9,10] High resource

settings use a commercialized oxygen blender for this purpose. These blenders are not only expensive—about US$1,000 each—but require that both oxygen and air are supplied from a pressurized source such as a wall supply or cylinders. Further, the pressure of both gases must be roughly equal in magnitude and at a pressure beyond the capability of most oxygen concentrators. Pressurized air is rarely available in resource-constrained settings as it has very few uses beyond mechanical ventilation. Therefore, the provision of blended oxygen is not available in most resource limited settings providing bCPAP.

Makerere University School of Public Health • Higher Degrees, Research and Ethics Committee •
Valid Thru:
04 MAY 2022

2.0 CHAPTER TWO: Literature review

Neonatal mortality

Over five million children under age five die annually worldwide - the majority in low resource settings.[11] The risk of death is highest in the first month of life, with 18 deaths per 1,000 live births globally, accounting for 2.5 million deaths overall.[11] Over the last 30 years, paediatric mortality has improved more quickly than neonatal mortality due to more rapid improvements in post-neonatal mortality.[12] Neonatal deaths are now responsible for nearly half (47%) of all childhood mortality. [11] Sub-Saharan Africa has the highest burden of paediatric mortality- 16 times the average mortality rate in high income countries. [11] In Uganda, neonatal mortality remains 28/1000 live births, and the largest cause of death is prematurity.[13] Accelerating implementation of available, cost-effective interventions for sick newborns is a factor into reducing this mortality.

Respiratory failure in neonates

Respiratory failure is the final common pathway in the most frequent causes of neonatal death: preterm birth, infection, and birth asphyxia.[11] Preterm babies in particular have a deficiency in surfactant in their lungs and have difficulty keeping them open following birth. There is increasing attention on the care of the sick and small newborn including increased development of interventions for respiratory failure, such as bubble continuous positive airway pressure (CPAP).[14]

Evidence for bubble CPAP

Bubble Continuous Positive Airway Pressure (bCPAP) is a simple, form of respiratory support that has been shown to decrease mortality in neonates with respiratory distress in both high and low resource settings.[15-17] Pressure delivery in bubble CPAP is due to the flow of air through a tube placed under water. The pressure is dependent on the depth of this tube. Bubbles emerging from the tube create oscillations which are trasmittted to the patient's lungs. Treatment with bCPAP has been associated with improved clinical outcomes compared with other forms of CPAP.[18,19]

Bubble CPAP is increasingly used in low and middle income countries.[20-22] In Uganda it has been associated with improved mortality among preterm infants.[23,24] A 2020 publication from Mbale reported, among patients <1500 grams birthweight, the mortality rate decreased from 39% to 27% after the initiation of bCPAP (44% reduction in mortality, OR 0.56, 95%CI 0.36 – 0.86, p = 0.01).[23] The site of our study, Kiwoko Hospital, in Nakaseke district, was the site of bCPAP implementation in 2012 and one of the first published case series of such use in Uganda.[24]

Bubble CPAP Devices

Bubble CPAP is strongly recommended for treatment of premature lung disease.[25] However there are limitations to available devices- commercialized bCPAP can cost over 10,000USD.[16] Lower cost commercialized bCPAP devices exist, however they cost 900 USD or more and low resource setings cannot afford enough units to serve the number of patients requiring bCPAP treatment.[26,27] The WHO recomends settings without access to commercialized devices create improvized bCPAP.[7] These improvised kits (Figure 2) and

Makerere University School of Public Health • Higher Degrees, Research and Ethics Committee • Valid Thru: 04 MAY 2022

described in section one), use spare parts that are commonly available in facilty settings and have allowed more settings to treat patients with bCPAP. Improvised kits have not been bench tested for reliability, and the design (using small bore oxygen tubing) varies considerably from those of commercialized designs. The need exists, therefore, for a low cost bCPAP design that has undergone testing to confirm it delivers accurate and appropriate pressure to a newborn.

Oxygen Safety

Oxygen is life saving for newborns and is included in the WHO model list of essential medicines.[28] Hower the use of 100% oxygen can be dangerous. Excessive oxygen use causes retinopathy of prematurity (ROP), blindness or visual impairment in preterm infants. This is one of the leading causes of blindness around the world, causing 32,200 cases in 2010.[29] Currently ROP is most prevelent in middle income countries as neonatal care has scaled-up without safe oxygen management.[29] Oxygen-associated blindess is now being reported in Africa, likely associated both with increased oxygen use as well as ability to follow-up and detect this retinal damange in tertiary neonatal units.[30-32]
Although 100% oxygen should never be used for bCPAP therapy, many resource limited sites implementing bCPAP have access only to 100% oxygen.[9] Blending oxygen with room air allows it to be titrated to the patient's oxygen saturations and decreases the risk of hyperoxia. Low cost systems for delivering blended oxygen are required for safe bCPAP use.[9]

Makerere University School of Public Health • Higher Degrees, Research and Ethics Committee •
Valid Thru:
04 MAY 2022

3.0 **CHAPTER THREE**: Statement of the problem, Justification and Conceptual framework

3.1 Problem statement

The use of bCPAP devices can reduce mortality related to respiratory failure in newborns. However, devices operated with 100% oxygen increase the risk of morbidity in survivors, due to oxygen toxicity. There is a critical need for a safe, rigorously tested, very low-cost bCPAP kit including oxygen blender to be made available to resource-constrained sites.

3.2 Justification of the problem

To address this need, PATH has developed a low-cost bCPAP kit which includes two fixed-ratio oxygen blenders (Figures 3 and 4) that do not require a source of pressurized air. These simple and inexpensive devices sit in-line from the oxygen source and dilute the flowing stream of oxygen with room air, obviating the need for a pressurized source of air. The oxygen may be supplied from either high-pressure sources or low-pressure sources such as oxygen concentrators. Each of the two blenders are single plastic parts, injection moulded from a biocompatible, medical-grade polyethylene resin. No electricity is required for the blenders to provide a stable and constant mix of air and oxygen over the full range of clinically relevant pressure and flows for newborns.

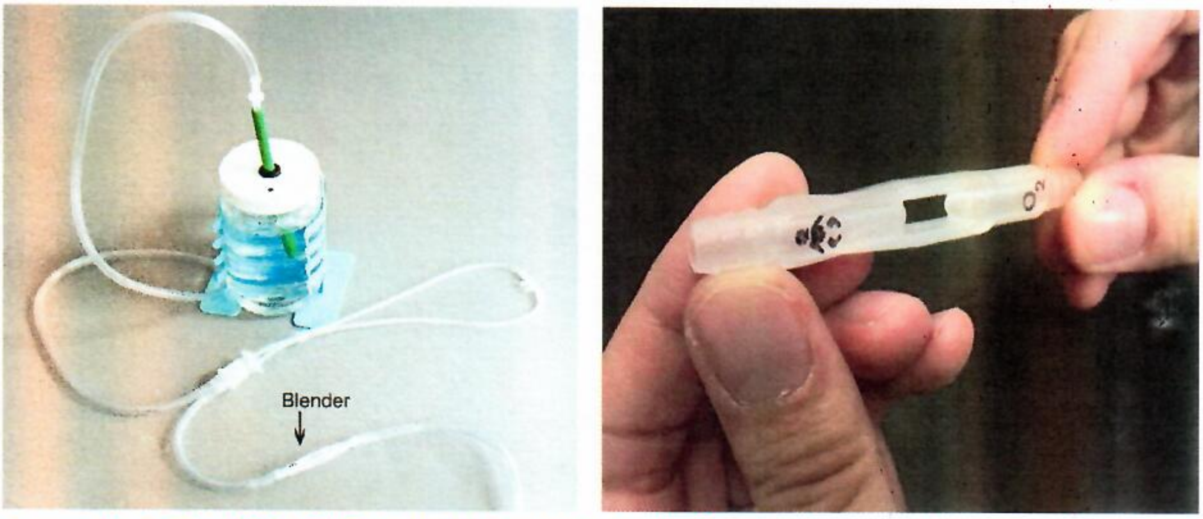

Figure 3. PATH BCPAP Kit with in-line blender (left) and close-up of oxygen blender (right)

Makerere University School of Public Health • Higher Degrees, Research and Ethics Committee •
Valid Thru:
04 MAY 2022

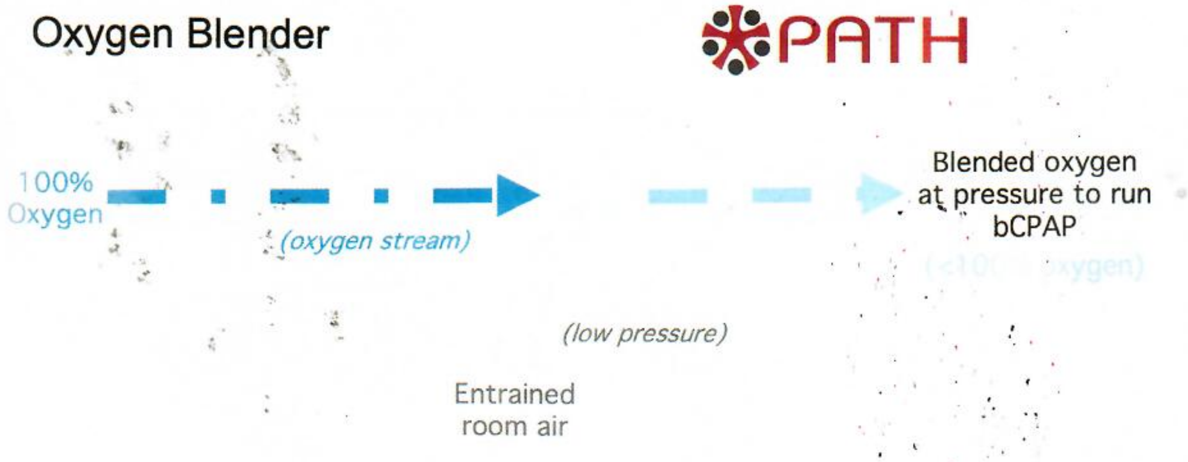

Figure 4. Mechanism of oxygen blender

Two fixed ratio blenders are available: a 'low' blend that provides 37% oxygen, and a 'high' blend that provides 60% oxygen. The actual ratio of oxygen and air necessarily depends on the concentration of oxygen supplied to the blender but remains constant and stable for any given input.

Both the technological characteristics and intended use of PATH's blenders are substantially equivalent to devices that are on the US market today. The mode of operation (air entrainment) has been employed by class 1 (low risk) oxygen diluter devices used for high-flow oxygen masks that have been commercially available since the 1970's. Oxygen blenders (class 2, moderate risk) have been commercially available for a similar period, and provide variable blends of air and oxygen for respiratory support requiring some pressure—such as CPAP. The PATH fixed-ratio blenders combine the technological characteristics of the former (fixed-ratio air entrainment), with the intended use of the latter (CPAP therapy).

In the USA, the PATH blenders would be considered a class 2 device and would follow the substantial equivalence regulatory pathway, otherwise known as a 510(k) or premarket notification. Typically, class 2 devices are not required to demonstrate clinical safety or efficacy as their technological characteristics are well understood and their intended uses are identical to those of devices already cleared. Characterization of the device's safety and performance can be demonstrated through performance testing of the flow, pressure and oxygen fraction. The verification test report for the bCPAP kit and blenders can be found in Appendix B.

We seek to conduct an early feasibility study (EFS), which is a limited clinical investigation of a device in development, typically before the device design has been finalized, for a specific indication (e.g., innovative device for an established intended use). The purpose of this study is to assess the operational feasibility, usability and acceptability of the device in a resource-constrained setting. Although there have been no prior studies in human populations with this device, oxygen blenders are a critical safety component of bCPAP therapy; providing unblended (100%) oxygen to premature infants can cause blindness, chronic lung disease and brain damage. The World Health Organization recommends that blended oxygen or compressed air—not pure oxygen—be used for these patients.[7]

Makerere University School of Public Health • Higher Degrees, Research and Ethics Committee • Valid Thru: 04 MAY 2022

Once this study is completed, the device labelling and design may be revised if needed and prepared for manufacture by Sinapi biomedical (Stellenbosch, South Africa). This low-cost bCPAP kit with oxygen blenders will allow resource-constrained sites to provide rigorously tested bCPAP therapy and blended oxygen to patients with reduced risk of morbidity from oxygen toxicity. This study is aligned with WHO priorities. In 2018, the WHO called for more research to be done to explore simpler, cheaper technology to improve supportive care of newborns who require oxygen, including safe bCPAP therapy—estimating that the introduction of a low cost bCPAP kit with blenders in central and district hospitals would prevent 178,000 neonatal deaths in Africa each year.[27,33]

3.3 Study Product

Overview

The PATH bCPAP kit with oxygen blenders is intended to provide respiratory support to a neonate in respiratory distress where higher level means of respiratory support are not available. (See Appendix A for product label and instructions for use). This product is a single-use device for the purposes of this study. No changes to device design will be made during the study.

PATH's fixed-ratio, in-line oxygen blenders are small, single-use, injection moulded devices intended to provide a stable blend of air and oxygen to a neonate on bCPAP therapy. (Instructions for use in Appendix A). A blender is inserted (in-line) into the inspiratory limb of a bCPAP respiratory circuit and works by entraining room air into a stream of pressurized oxygen (based on the Bernoulli principle), producing a gently pressurized and blended gas that is delivered to the neonate (see Figures 4 and 5). The device provides a fixed ratio of air and oxygen that is consistent over the entire range of clinically relevant pressures (3 to 10 cmH_2O) and remains consistent despite changes in flow (1 to 10 LPM). The blend is stable within ± 5% of the blender's nominal blend-ratio when used with 100% oxygen source, irrespective of the flow and pressure from the oxygen source, or the flow and pressure of the blended gas delivered to the neonate.

Makerere University School of Public Health • Higher Degrees, Research and Ethics Committee • Valid Thru: 04 MAY 2022

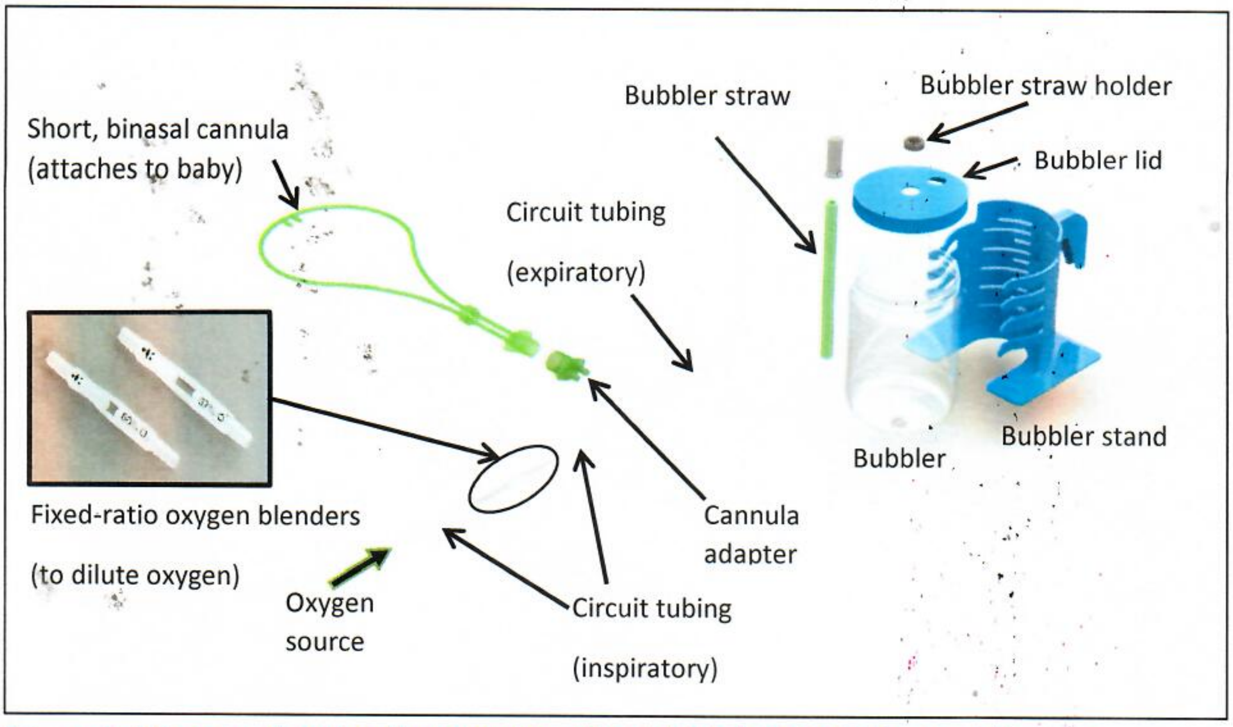

Figure 5. Diagram showing the components of the PATH bCPAP circuit (tubing, cannula and bubbler) as well as placement of PATH oxygen blenders

Flow and pressure of the blended gas are adjusted in the same manner as respiratory circuits containing no blender (i.e., by adjustment of the flow of gas entering the system and depth of the bubbler straw within the bCPAP bottle, respectively). To change the blend ratio, the blender is simply replaced with another to achieve a higher or lower percentage of oxygen delivered. Currently two fixed-ratio blenders have been developed; a "low blender" with a blend-ratio of 37% and a "high" blender with a blend-ratio of 60% (see Figure 6). The rationale for having two blends for this study is two-fold: (1) more blend ratios would complicate the decision-making process by the healthcare worker and (2) more blend ratios could increase the amount of time spent swapping out blenders. However, additional blend ratios between 37% and 60% can be produced if this study suggests that more blending options would be of benefit.

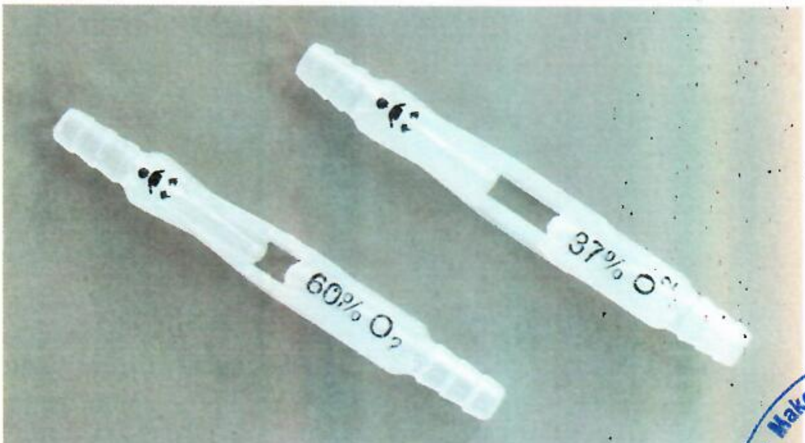

Figure 6. 37% and 60% oxygen blenders

Makerere University School of Public Health
Valid Thru:
04 MAY 2022
Higher Degrees, Research and Ethics Committee

In facilities with access to both pressurized air and oxygen, commercially available, fully adjustable oxygen blenders can be used to adjust the blend ratio between 21% (room air) and 100% oxygen. Both air and oxygen must be provided at equal pressures of 50 psi in order for the blender to perform accurately. For this reason, commercially available, adjustable blenders cannot be used with oxygen concentrators because the pressure generated by these devices is too low.

In contrast, the PATH fixed-ratio oxygen blenders do not depend on a source of pressurized air, as air is drawn from the room itself. Furthermore—and unlike a commercially available, adjustable oxygen blender—the oxygen can be provided by an oxygen concentrator. Choice of blend ratio is limited, however, to the discrete fixed-ratio blenders at hand. As noted earlier, changes in blend ratio are accomplished by replacing one fixed-ratio blender for another of the desired percentage of oxygen.

Design & Manufacture

Sinapi biomedical is an ISO 13485:2016-certified medical device manufacturer based in Stellenbosch, South Africa. They were responsible for production, labelling, and packaging of the fixed-ratio oxygen blenders intended for use in the study. Sinapi has 18 years of experience in production of medical devices that are distributed in both South Africa and across Europe. bCPAP kits and blenders were produced in Sinapi's production clean room environment and each kit and blender were visually inspected and tested for performance (oxygen fractionation) to ensure proper performance. Results of this testing can be found in the verification test report (Appendix B)

PATH's bCPAP kit and blenders are an investigational device, produced in South Africa. It does not fall under the jurisdiction of the US Food and Drug Administration (FDA), but given the lack of regulation for investigational devices in Uganda (the location of the study), FDA guidelines have been used as a best practice. In the US, the pathway for marketing authorization is based on the potential risk a device poses to human safety. In the US, variable-blend oxygen blenders are classified as class II devices and would be cleared based on the 510(k) (substantial equivalence) pathway. If a 510(k) pathway were followed, the predicates for the oxygen blender would be as follows:

- §868.5330 (Product code: BZR; Class 2): A breathing gas mixer is a device intended for use in conjunction with a respiratory support apparatus to control the mixing of gases that are to be breathed by a patient. **Predicates**: Precision Blender; Bird Air/Oxygen Blender.
- §868.5600 (Product code: BYF*; Class 1): A venturi mask is a device containing an air-oxygen mixing mechanism that dilutes 100 percent oxygen to a predetermined concentration and delivers the mixed gases to a patient. **Predicates**: Salter air entrainment oxygen mask.

Class II devices generally do not require clinical data to be generated in order to obtain marketing authorization, as their safety and effectiveness is well documented in the literature and the mechanism of action well understood.[34] Fixed-ratio oxygen blenders (entrainment devices) are classified as class I (low risk) devices, but are indicated for use with adult, high flow masks. Because our fixed-ratio oxygen blender is intended for use with neonates, we

Makerere University School of Public Health • Higher Degrees, Research and Ethics Committee •
Valid Thru: 04 MAY 2022

have taken the most conservative approach and assumed the 510(k) [i.e. class II] pathway for marketing authorization would be required if the device were to be marketed in the United States. See Appendix B for Substantial Equivalence Table.

The fixed-ratio oxygen blender is in the final stages of development. The design has been rigorously tested at the bench to ensure its safety and performance. Table 1 lists the testing that has been conducted on the 37% and 60% blenders. Table 2 lists the composition and manufacturer of the bCPAP kit components.

Table 1. Status of Fixed-Ratio Oxygen Blender Verification Tests

Verification test	Criteria	Status
Blend stability*	Stable blend (±5% of nominal FiO_2)	Passed
Temperature & humidity	Stable blend at 30 °C and 70% relative humidity	Passed
Noise	< 80 dBA	Passed
Shelf life†	Stable blend at 1 year	Pending
Biocompatibility	Per ISO 18562-2:2017	Passed

**Blend stability testing has been conducted under worst case conditions of flow, pressure, temperature and humidity.*

† Shelf life testing is based on accelerated aging test results at the time of writing and is intended to ensure performance of the device for the duration of the study. However, real-time shelf life testing is ongoing, and it is expected that the actual shelf life of the device will be confirmed to be much longer.

Table 2. Components of the bCPAP kit

Component	Material	Manufacturer
Bubbler stand	Acrylonitrile Butadiene Styrene (ABS)	3D printed (custom)
Cannula adapter*	Polypropylene (PP)	Protolabs (custom)
Blenders (37% & 60%)*	Polypropylene (PP)	Protolabs (custom)
Short, binasal cannula**	Polyvinylchloride (PVC), silicone	Neotech
Bubbler straw holder	Nitrile rubber	McMaster
Bubbler straw	High density polyethylene (HDPE)	McMaster
Bubbler & bubbler lid	Polyethylene terephthalate (PET), high density polyethylene (HDPE)	McMaster
Circuit tubing*	Polyvinylchloride (PVC), thermoplastic elastomer (TPE)	McMaster

* *These components are not in direct contact with the patient but are in contact with the respiratory gases that flow to the patient.*

*** This component is in contact with the patient's nares. There are multiple sizes provided to fit the range in neonatal nares sizes.*

The nasal cannula is the only component of this device that will be in direct contact with the patient. The Neotech RAM Cannula used in the PATH bCPAP kit (see: https://www.neotechproducts.com/product/neotech-ram-cannula/#) is CE marked and FDA registered so it meets the ISO 10993 standard for biocompatibility. The Neotech RAM

Makerere University School of Public Health • Higher Degrees, Research and Ethics Committee • Valid Thru: 04 MAY 2022

Cannula is not manufactured with DEHP, which is the most common phthalate plasticizer in medical devices.

The purpose of this study is not to confirm the safety or efficacy of PATH's bCPAP kit and blenders, as this has already been established by bench testing against the predicate. Rather it is to evaluate the feasibility of using fixed-ratio blenders to reduce the amount of oxygen delivered to neonates to meet oxygen saturation targets as indicated by pulse oximetry in <u>instances where the only existing choice is to provide 100% oxygen</u>. Exceeding oxygen saturation targets by use of 100% oxygen is extremely harmful and can lead to permanent eye, lung and brain damage, especially for those neonates born prematurely.

Makerere University School of Public Health • Higher Degrees, Research and Ethics Committee •
Valid Thru:
04 MAY 2022

4.0 **CHAPTER FOUR**: Research questions, hypothesis and objectives

4.1 Hypothesis

We hypothesize that the PATH bCPAP kit with oxygen blenders will be easy to use by healthcare workers treating neonates in respiratory failure and allow the patient to receive less than 100% oxygen when no other standard blending mechanism is available.

4.2 General objective

The purpose of this research is to conduct an early feasibility study (EFS) of PATH's bCPAP kit including use of PATH oxygen blenders when no other standard blending mechanism is available.

4.3 Specific objectives

Primary Objectives:

1. To assess the <u>operational feasibility</u> of using the PATH bCPAP kit including, when appropriate, in-line oxygen blending on neonatal patients.
2. To assess the <u>usability and acceptability</u> of the PATH bCPAP kit with oxygen blenders by healthcare workers.

Secondary Objective:

1. To report clinical characteristics, demographics and outcomes of patients treated with the PATH bCPAP kit and blenders in a newborn care unit in rural Uganda.

Makerere University School of Public Health • Higher Degrees, Research and Ethics Committee •
Valid Thru:
04 MAY 2022

5.0 **CHAPTER FIVE:** Methodology

5.1 Study design

This is an early feasibility study to evaluate the device's human interface and its impact on operator technique. As such, it is a limited clinical investigation. The study will help to obtain insights into: successful use of PATH's bCPAP kit with oxygen blenders, operator technique challenges, human factors (such as use errors and difficulties comprehending procedural steps) and patient characteristics that may impact device performance (anatomical limitations). The experience obtained from the study will be used to identify appropriate modification to the use procedures and/or the device as needed.

The study will take place in a newborn care unit at Kiwoko hospital, Uganda with experience using bCPAP and blended oxygen. It will use mixed methods, employing both quantitative and qualitative approaches. We will conduct prospective data collection of the usability and acceptability of the PATH bCPAP kit with oxygen blenders through observation of and in-depth interviews with healthcare workers, as well as prospective data collection of the clinical characteristics and outcomes of patients treated with the kit and blenders.

5.2 Study site

The study site is the newborn care unit of Kiwoko Hospital, a rural, private, not-for-profit, secondary level care hospital that acts as a referral centre for three districts in central Uganda. Neonates below 44 weeks corrected gestational age are admitted to the newborn unit. Half of the admissions are born at the hospital and the remaining half are born at home or transferred from another facility. This unit is accredited for 38 beds, and during 2018 it provided care for 1,223 newborns.(11) There are around 36 nurses on staff in the newborn unit with four to six of them on duty each eight hour shift, as well as one to two assigned general practice or paediatric physicians. Electricity is constantly available with the back-up of a generator. The unit provides thermoregulation, intravenous hydration, continuous or intermittent nasogastric feeding, phototherapy, antibiotics, oxygen therapy via concentrator and intermittent or continuous pulse oximetry. Neither mechanical ventilation nor surfactant is available in the unit. In Kampala, where mechanical ventilation and surfactant are available, the cost is prohibitively expensive for the population served by Kiwoko Hospital.

Providing bCPAP therapy with improvised bCPAP kits is the standard of care for respiratory failure in the unit since 2012. The existing Kiwoko bCPAP is assembled from spare parts and donated nasal cannulas.(12) This improvised kit will be referred to as the Kiwoko bCPAP kit for purposes of this protocol.

Patients at Kiwoko have intermittent access to blended oxygen while on bCPAP therapy. This is provided by the combination of flow from an oxygen concentrator and an air compressor. A connector, shaped like the letter Y, combines the flow from the two sources to provide blended oxygen and air to the patient. (Figure 7.). Adjusting the ratio of flows from each determines the amount of oxygen delivered. Unfortunately, these air compressors cannot be sourced in-country and have been brought in from the United States. Higher demand for clinical use of the compressors dictates the devices run continuously. Despite regular maintenance, these devices often breakdown and repair parts are difficult to source.

Makerere University School of Public Health • Higher Degrees, Research and Ethics Committee • Valid Thru: 04 MAY 2022

The system is not sustainable nor scalable. The number of patients who can receive blended oxygen is limited by the availability of these compressors—typically 0-2 are functioning at one time. This can result in some babies receiving bCPAP therapy with up to 100% oxygen.

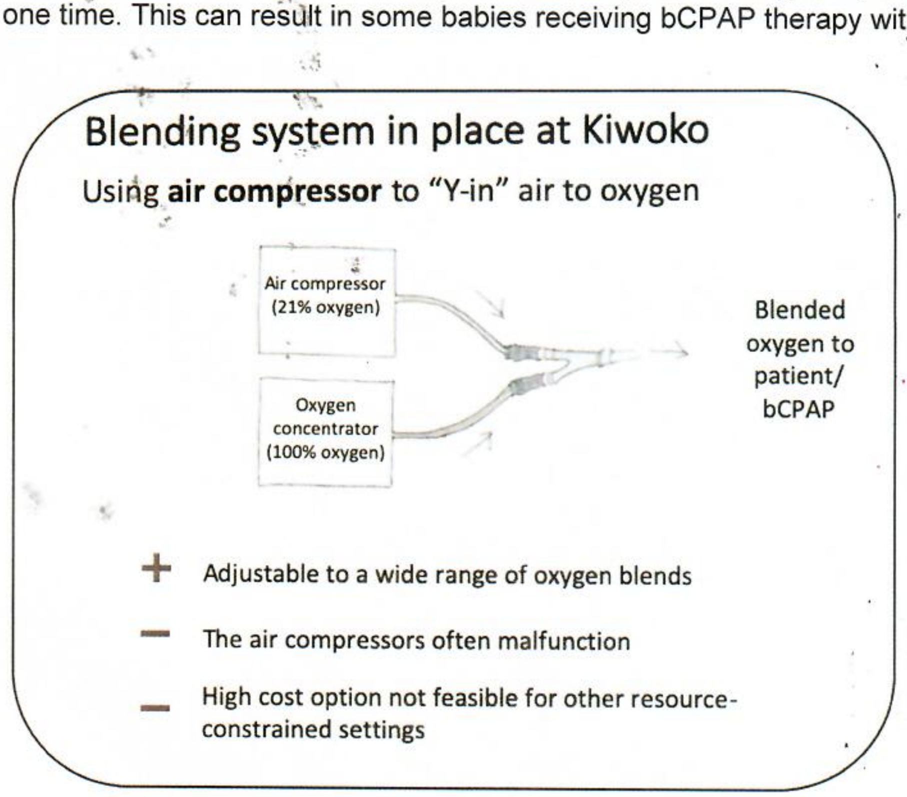

Figure 7. Oxygen blending system in place at Kiwoko Hospital. This system can provide a more precise blend of oxygen than the PATH blender but, due to reliance on a functioning air compressor, is very limited in its availability to serve patients.

5.3 Sample size

As this is an early feasibility study with no control or comparator arm, the sample size will be purposeful and not hypothesis driven.

Patients:

The sample size for patients will allow for reasonable frequency of use of the device by healthcare workers and at the same time for risk mitigation in line with an early feasibility study.

During the period of study, up to 40 patients will be treated with the PATH bCPAP. **This maximum of 40 patients in this study is selected to ensure that we will achieve our required sample size of 15 infants who are treated with the PATH bCPAP kit and blender.** All enrolled patients will receive treatment with the PATH bCPAP kit (cannula, tubing, bubbler bottle), although not all may require the use the PATH blender. Patients may have two methods of blending available during the study according to the following decision logic (Figures 8 and 9):

- **An air compressor will be used as first choice if available**. The number of patients in this scenario is dependent on the availability of functional air compressors; see Study Site section 5.2 for further details.

Makerere University School of Public Health • Higher Degrees, Research and Ethics Committee • Valid Thru: 04 MAY 2022

- When a functional air compressor is not available, the patient will receive treatment with the PATH blenders. Our aim is to treat 15 patients using the PATH blenders. No additional patients will be enrolled in the study after this target has been achieved.

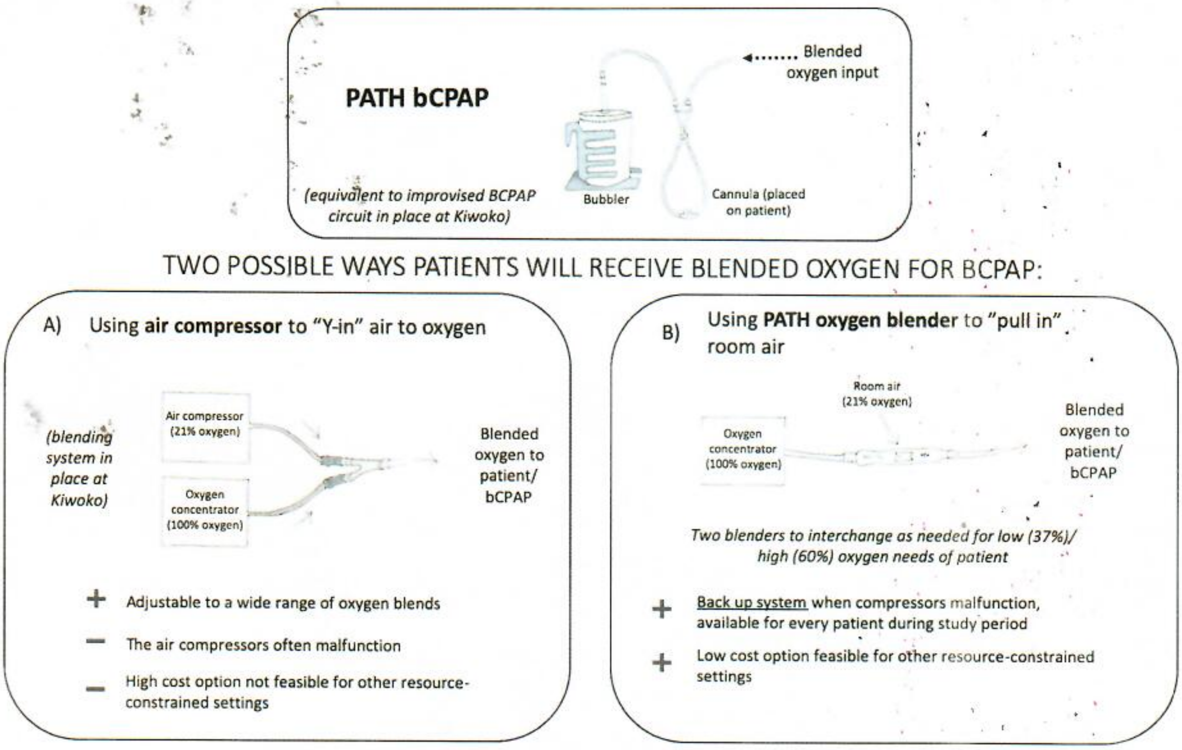

Figure 8. Methods of oxygen blending during study. The study may enrol up to 40 patients on the PATH bCPAP kit to ensure a subset of 15 patients will also use the PATH oxygen blender (B).

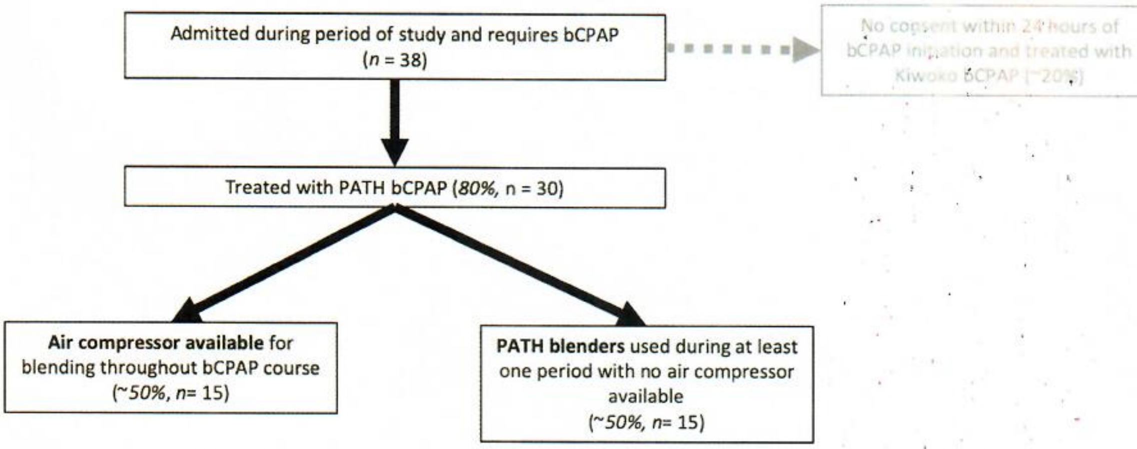

Figure 9. Flowchart of expected patient recruitment (percentages and n's estimated). A total of 30+ enrolled patients treated with PATH bCPAP may be required to ensure the target of 15 patients are also treated with the PATH blenders.

Makerere University School of Public Health • Higher Degrees, Research and Ethics Committee • Valid Thru: 04 MAY 2022

Sample size for patients is being estimated knowing that at Kiwoko Hospital, an average of 12 patients are treated with bCPAP therapy per month[35], per the Table 3 below.

Table 3. Expected recruitment and PATH blender use based on study length and functional compressors.

Study length (in months)	Expected number of patients on CPAP	Enrolled patients with 80% consent	Patients with use of PATH blender (air compressor not available ~50% of time)
1	12	9	4
2	24	19	10
**3**	**37**	**29**	**15**
4	49	39	20
5	61	48	24

Healthcare workers:

Bedside observations: We will observe the care of bCPAP patients with or without PATH blender use provided by healthcare workers in the newborn care unit. This will include all or most of the 40 permanent healthcare workers in the newborn care unit who care for patients on bCPAP therapy and have consented to participate in the study.

Qualitative interviews: Up to 15 of those same healthcare workers who were observed using the PATH bCPAP kit with blenders will be interviewed within the first five days of their first use of the PATH bCPAP kit with blenders. Additionally, five of those healthcare workers that used the PATH bCPAP and blenders kit three or more times during study period will be interviewed again towards the end of the study to probe if their experience changed with familiarity of use.

5.4 Study population

Patients:

Inclusion criteria *for use of PATH bCPAP and oxygen blenders*: Newborns <44 weeks post menstrual age with respiratory failure requiring treatment with bCPAP therapy during the period of study and whose parent or legal guardian consented to participation in the study, within 24 hours of bCPAP initiation.

Exclusion criteria: No parental nor legal guardian consent to participation in the study.

Patients will be withdrawn from the study if their parent or legal guardian withdraws consent. If this occurs, *they will no longer be treated with PATH bCPAP nor blenders.*

Patients admitted to the Kiwoko Hospital newborn unit are <44 weeks post menstrual age, primarily neonates (<28 days of age) in the first 3 days of life. Approximately 50% of the population admitted to the newborn unit is preterm (born before 37 weeks gestation). Half of admissions were born outside Kiwoko [35,36]. The decision to initiate bCPAP therapy is

Makerere University School of Public Health • Higher Degrees, Research and Ethics Committee
Valid Thru: 04 MAY 2022

made according to unit guidelines and physician/nurse discretion based on assessment of the degree of respiratory failure (See Appendix C). Fifteen percent of patients admitted to the Kiwoko newborn unit are treated with bCPAP therapy, with a birthweight distribution as shown in Table 4.[35] Unit guidelines specify criteria for adjustment of bCPAP pressures, oxygen blends and weaning off bCPAP support. Median length of bCPAP therapy is 3 days. The baseline mortality for patients treated with bCPAP in the Kiwoko unit is 34%.[35]

Table 4. Anticipated PATH bCPAP kit use by birthweight category based on average Kiwoko bCPAP kit use in the Kiwoko newborn unit

Birthweight Category	Percent of overall bCPAP use expected of this category[37]
<1kg	8%
1-1.4kgs	22%
1.5-2.4 kgs	33%
2.5-4kgs	35%
>4kgs	2%

Patients will be withdrawn from the study if their parent or legal guardian withdraws consent. If this occurs, they will be changed to the previous Kiwoko bCPAP kit and blending will be provided via air compressor when available. If there are no functioning air compressors at that time, blending will not be available, and bCPAP therapy will be provided by oxygen concentrator only.

Healthcare workers:

Inclusion criteria:

- Healthcare workers (nurses and midwives) who care for patients on bCPAP therapy with or without blenders at Kiwoko's newborn unit during the period of study, and who provided informed consent to be observed and interviewed

Exclusion criteria: Healthcare workers that did not provide informed consent to be observed or interviewed.

5.5 Study procedure

Patients:

During the study, all patients admitted to the newborn care unit with respiratory failure will be evaluated for treatment with bCPAP per unit standards. All patients that meet treatment criteria will receive bCPAP. Patients for whom consent is available will be enrolled and start bCPAP therapy with the PATH kit. Patients for whom consent is not given or not available will start Kiwoko bCPAP. Patients who began bCPAP therapy with the Kiwoko kit and obtain consent within 24 hours of starting their bCPAP therapy may switch from Kiwoko to PATH bCPAP (see description of this process below). Patients with >24 hours of Kiwoko bCPAP therapy are no longer eligible for enrolment.

Makerere University School of Public Health
Valid Thru:
04 MAY 2022
Higher Degrees, Research and Ethics Committee

The PATH bCPAP kit is comprised of the same components as the Kiwoko bCPAP kit currently in use (nasal cannula, connectors, tubing, bottle - see section 8.0 "products"). Set-up time is expected to be comparable to the Kiwoko bCPAP kit. There is no significant difference in performance between the PATH and the Kiwoko bCPAP kits in the absence of the blenders. The only new feature of the PATH bCPAP kit is the option of using the fixed ratio oxygen blenders. The PATH blenders enable the bCPAP system to provide less than 100% oxygen without the use of an air compressor. The PATH bCPAP kit with blenders is single use and will be discarded after use on one patient. Standard of care will not change with the use of the PATH bCPAP kit with or without blender—the unit will continue to administer bCPAP therapy in accordance with unit guidelines (see Appendix C) and physician/nurse discretion. If parents decline consent, the patient will be treated using the Kiwoko bCPAP kit that was previously in use. Patients may also be photographed, video or audio recorded if parents/ legal guardians specifically consent to this.

Patients who start bCPAP therapy using the Kiwoko bCPAP kit, and then have consent obtained in the first 24 hours of treatment, will switch to the PATH bCPAP kit. The PATH bCPAP kit will be assembled prior to the switch over, allowing the circuits to be easily exchanged by disconnecting the tubing to the cannula and connecting the new PATH kit tubing. The cannula (and tape affixing it to the patient's face) will not be changed as both Kiwoko bCPAP and PATH bCPAP use the same cannula. This switchover will occur very quickly- the brief interruption of pressure to the patient is similar to what occurs with other procedures on bCPAP, such as suctioning or cannula adjustments.

During the study, oxygen blending for patients treated with PATH bCPAP will occur by the following two methods- and will be used in this order of preference (see Figure 8 "Methods of oxygen blending during study" in section 5.3):

1.) blending via air compressor (standard of care in unit and used when available)
2.) blending via PATH blender (when no compressor available)

Some patients will receive blending via both systems during their bCPAP treatment course due to changing availability of the air compressor (i.e. if it stops working or one becomes available as another patient has stopped using it).

Patients not enrolled in the study will continue to have blending available by standard of care (use of air compressor when available). When the air compressors are not available the patients are treated with the oxygen concentrator which emits the closest oxygen requirement to meet their needs (see explanation below under "oxygen use"). Patients may receive 100% oxygen if there are no concentrators emitting lower percentages. As identified earlier, the use of 100% oxygen is not recommended as it increases the risk of morbidities such as blindness, chronic lung disease and brain injury.

bCPAP Use

The unit will continue to use bCPAP therapy in accordance with unit guidelines (see Appendix C) and physician/nurse discretion. Kiwoko's newborn unit currently provides a high standard of care which will not change during the period of study.

Makerere University School of Public Health • Higher Degrees, Research and Ethics Committee • Valid Thru: 04 MAY 2022

Current bCPAP therapy standard of care in Kiwoko's newborn care unit:

- Nurse to patient ratio is usually 1:4.
- Continuous oxygen saturation monitoring when initially placed on bCPAP therapy.
- Patient assessment every 15 to 30 minutes in the first hour after being placed on a bCPAP kit.
- Patient assessment including vital signs every 3 hours after condition stabilizes.
- Patient who remains unstable is monitored more regularly as needed until stable.
- In the case where there is an inadequate number of oxygen saturation monitors, a baby with stable vital signs who is close to coming off bCPAP therapy may receive intermittent spot checks for oxygen saturations.

Additional care offered during the study:

- Additional oxygen saturation monitors will be provided to the unit for this study. This will allow all bCPAP therapy patients to receive continuous monitoring throughout their entire course of treatment with bCPAP.
- Research assistants will notify staff if the bCPAP with or without blenders is set-up incorrectly or they have other concerns about patient safety.

Oxygen Use

In the Kiwoko newborn care unit, oxygen concentrators are used for the delivery of oxygen. They are tested regularly for the amount of oxygen they provide, as older concentrators often provide a lower concentration of oxygen. During the study, oxygen concentrators will be tested weekly and labelled with the percentage of oxygen they produce.

Patients will receive continuous pulse oximetry monitoring while on bCPAP therapy in order to titrate oxygen according to the patient's needs. However, oxygen saturations of sick infants can fluctuate frequently, making oxygen adjustments a challenge. There is a delicate balance between too little and too much supplemental oxygen exposure, as both can lead to harmful conditions, especially in premature infants. Consistent with WHO recommendations for oxygen use in neonates, the saturation goals are defined in the Table 5 below.(15) It is standard to set the alarm limits to 2% below and 2% above the target range.

Table 5. Target oxygen saturation ranges based on gestational age

	SpO2 target range (%)	Monitor Alarm Setting (%)	
		LOW	HIGH
Less than 32 weeks gestation or <1250 grams	88 – 95*	86	97*
Equal or greater than 32 weeks gestation <u>and</u> >1250grams	90 – 95*	88	97*

** For any infant in room air (no supplemental oxygen), the high monitor alarm can be set at 100%.*

Makerere University School of Public Health • Higher Degrees, Research and Ethics Committee •
Valid Thru:
04 MAY 2022

Nurses will titrate oxygen based on saturations as is current standard of care per Table 6 below:

Table 6. Bedside actions based on patient oxygen saturations

Patient Oxygen Saturation	Action
Any time oxygen saturation falls below SpO2 target range	• Assess infant for problems and reposition as needed<br>• Troubleshoot bCPAP system (check connections, nasal interface, assess for presence of secretions)<br>• Check pulse oximeter sensor and reposition or replace as needed
Oxygen saturation is still below SpO2 target range (but still at or above 80%) for 5 minutes<br>or<br>Oxygen saturation falls below 80% for 1 minute	• Increase percentage of oxygen delivered<br>• Monitor saturations closely for 10-15 minutes after making the change
Oxygen saturation drops severely and the baby needs emergency attention	• Increase oxygen percent immediately
Oxygen saturation above SpO2 target range for 5 minutes	• Decrease percentage of oxygen delivered<br>• Monitor saturations closely for 10-15 minutes after making the change

There are times when the air compressors on the unit may not be functioning or are unavailable due to high demand. This creates two possible scenarios for oxygen blending during the study (Figure 6, Tables 7 and 8).

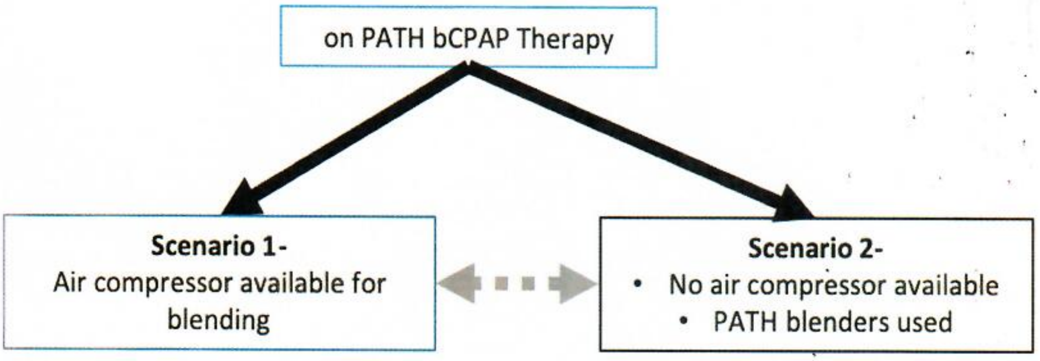

Figure 10. Scenarios of oxygen blending for enrolled patients during study. Patient's blending scenario may change throughout course due to changing availability/functioning of air compressors.

Makerere University School of Public Health • Higher Degrees, Research and Ethics Committee • Valid Thru: 04 MAY 2022

Table 7. Scenario 1- Adjusting oxygen delivery in current standard of care (when air compressor is available):

Percent Oxygen Required	How to Deliver
Lowest possible	All flow from air compressor<br>No flow from the oxygen concentrator
Low	Minimal flow from oxygen concentrator, most from air compressor
Middle	Flow split between air compressor and oxygen concentrator
High	Most flow from oxygen concentrator, minimal from air compressor
Highest possible	All flow from oxygen concentrator<br>No flow from the air compressor

Table 8. Scenario 2- Adjusting oxygen delivery with PATH blender (when no air compressor is available):

Percent Oxygen Required	How to Deliver
Low	Place 37% blender^ in line
Higher	Place 60% blender^ in line
Highest possible	All flow from oxygen concentrator<br>No blender use

^ The actual percentage of oxygen delivered to patient will be dependent on the concentration of oxygen provided by concentrator.

All patients, whether enrolled in the study or not, will receive standard care after bCPAP treatment. There will not be any study-related treatment or follow up provided at the end of this study. Patients discharged from the newborn care unit are offered follow-up visits free of cost at Kiwoko Hospital as is the standard of care.

Study Costs

All neonates who are admitted to the NICU and require bCPAP therapy can access it regardless of the parent's financial means. The hospital charges a base rate for admission, which is not affected by length of stay, nor basic treatments provided - bCPAP therapy is considered a part of basic treatment and is not an extra charge. At the time of discharge, patients are required to pay their bill. Families without financial means to pay their bill at discharge are considered for financial aid from the hospital.

5.6 Study variables

See Table 10 in Section 5.8.2

5.7 Data collection

Healthcare workers:
From the onset of the study, consented healthcare workers will be observed as they set up and use the PATH bCPAP kit with or without the oxygen blenders. If they have consented, healthcare workers may also be photographed, video or audio recorded. Observations conducted across the study period will ensure that different levels of familiarity with use of the blenders are observed.

- Right after the observation (or the next day if not enough time available), the healthcare workers will be asked to complete a usability scale related to their experience with the PATH bCPAP kit with or without oxygen blenders. We will collect data from healthcare workers in all 15 cases of use of the PATH bCPAP kit with the blenders.
- Up to 15 of those same healthcare workers who were observed using the PATH bCPAP kit with the blenders will also be interviewed within the first five days of having used the PATH bCPAP kit and blenders for the first time.
- Additionally, five of those healthcare workers that used the PATH bCPAP kit with blenders three or more times during study period will be interviewed again toward the end of the study, to probe if their experience changed with familiarity of use.

Data Collection:
A quality assurance activity will take place the week prior to the study to ensure HCW are following protocols re: oxygen blending with the existing Kiwoko bCPAP kit. No study data will be collected at this time.

Table 9. Study Timeline

Timeline	Activity
2 weeks before study start	Train In-Unit Research Assistants
2 weeks before study start	Train Data Team on REDCap Data Entry
2 weeks before study start	bCPAP and blending training for healthcare workers (education on PATH bCPAP kit and blender)
1 week before study start	Quality Assurance with Kiwoko bCPAP kit
**Study start**	Change to PATH bCPAP/blender and start data collection
Throughout data collection	Initial interviews with HCW after their initial use of PATH bCPAP kit/blender
3-4 months into data collection	Repeat interviews for selected healthcare workers with more familiarity of use of PATH bCPAP kit/blender
4 months total of data collection	Study end (3.5 months of PATH kit use)

5.7.1 Training

In order to standardize care, nurses and physicians working in the newborn care unit will receive mandatory refresher training two weeks prior to start of data collection. Refresher training will include clinical presentation of respiratory failure and providing treatment with

Makerere University School of Public Health • Higher Degrees, Research and Ethics Committee • Valid Thru: 04 MAY 2022

bCPAP. They will also receive training in the use of the PATH bCPAP kit, including the blenders. Healthcare workers who complete training will be recorded in a list and these are the only providers who will care for PATH bCPAP patients during the period of study. It is assumed the PATH bCPAP kit with oxygen blenders will be no more time intensive than the current system to set up or use, and there will be no additional charting requirements for the nurses.

To avoid disrupting clinical care of patients and to increase data quality, research assistants will be present 24 hours a day, all days of the week, at the bedside of patients receiving bCPAP therapy with or without PATH blenders during the study period. Research assistants will be nursing students trained to record clinical data points of patients on bCPAP therapy. They will serve in a purely observational role and will not provide patient care, nor make any modifications to treatment.

5.7.2 Tools

Makerere University School of Public Health • Higher Degrees, Research and Ethics Committee •
Valid Thru: 04 MAY 2022

See "Data Collection Forms" in Appendix D, these include:

- Patient Data Form 1 (Demographics and Admission Clinical Data)
- Patient Data Form 2 (Clinical Course and bCPAP Settings)
- Concentrator FIO_2 Output Form
- Newborn Unit Shift Form
- Adverse Event Record
- Unanticipated Adverse Device Effects (UADE) Event Record
- Healthcare Worker (HCW) Usability Form
- Healthcare Worker (HCW) Observation of bCPAP With or Without Blenders Set-up Form
- Healthcare Worker (HCW) Initial Interview Guide
- Healthcare Worker (HCW) Follow-Up Interview Guide

Data will be collected on the forms at the following timepoints:

Table 10. Patient Specific Data Collection Timing:

Form	Event					
	**bCPAP initiation**	**Blender start or change**	**bCPAP pressure change**	**bCPAP stopped**	**Patient discharged/. deceased**	**Unanticipated Adverse Device Effect or Adverse Event**
Healthcare Worker (HCW) Observation of bCPAP With or Without Blender Set-Up	**X**					
Patient Data Form 1 (Demographics	**X**			**X**	**X**	

and Admission Clinical Data)						
Patient Data Form 2 (Clinical course and bCPAP settings)	X	X	X	X		
Adverse Event Record						X
Unanticipated Adverse Device Effects (UADE) Event Record						X

Table 11. Unit/Equipment Specific Data Collection Timing:

	**Each Shift**	**Each Week**
Newborn Unit Shift Form	X	
Concentrator FIO_2 Output Form		X

Table 12. Nursing Specific Data Collection Timing:

	**With Each PATH bCPAP initiation**	**Initial Interview**	**Final Interview**
HCW Initial Interview Guide		X	
HCW Follow-up Interview Guide			X
HCW Usability Form	X		

Makerere University School of Public Health • Higher Degrees, Research and Ethics Committee •
Valid Thru:
04 MAY 2022

5.8 Data management and analysis

5.8.1 Data management

All quantitative data will be collected using case report forms and entered in REDCap software. The software will be programmed with internal checks to ensure completeness and logical entries. Data will be later transferred to Stata V.15 for analysis.

Qualitative data analysis: paper transcripts, files of data and audio recordings from in-depth interviews will be kept securely under key and lock. Audio recordings will be downloaded and stored in a password protected computer and transcribed. A codebook with code definitions and a data analysis plan will be developed before the coding exercise starts. As a quality control measure, about 10 primary documents will be double coded (manually and electronically). Transcripts will then be exported into NVivo software for electronic coding and analysis using content thematic approach guided.

5.8.2 Data analysis

Table 13 presents a summary of variables, data sources and analysis plan. We will evaluate operational feasibility by analysing the domains below; each domain uses information from mixed data sources. We will create an index measure for each domain, all of which will be weighted equally. We will sum these index measures to determine overall operational feasibility.

- Ease of correct use: Median set-up time, frequency of correct set up, time to change blender, types and frequencies of user errors, frequency of changes to blend provided per day, frequency of bCPAP level adjustment per day
- Integration into service delivery: Count of patients treated with PATH bCPAP, Frequency of blending via air compressor and PATH blenders, Frequency of known bCPAP complications
- Confirmation of device functionality: Median and IQR of: FIO2 administered by PATH blenders (determined by blender used and FIO2 provided by concentrator), level of bCPAP (and min/max), total air/oxygen flow to bCPAP device (subset)
- Concentrator output: Ability to determine FIO2 delivered to patient
- Device malfunction: Frequency of device malfunctions with and without adverse events

Table 13. Summary of Relevant Data Analysis for Study Objectives

Objective	Variables	Source of Data	Statistical analysis
Objective 1: To assess the <u>operational feasibility</u> of using the PATH bCPAP kit including, when appropriate, in-line oxygen	Observed use of kit by HCWs including completion of set-up steps, time to set-up, description of difficulties	• Healthcare Worker (HCW) Observation of bCPAP With or Without Blender Set-Up	We will determine frequency of various parameters/variables collected on the use anc set-up of the kit<br><br>Mean, standard deviatio medians and interquartil ranges will be used for

Makerere University School of Public Health • Higher Degrees, Research and Ethics Committee • Valid Thru: 04 MAY 2022

blending on neonatal patients		• Patient data form 2 (clinical course and bCPAP settings)<br>• Concentrator FIO2 output form	continuous variables ba[illegible] on their distribution
Objective 2: To assess the <u>usability</u> of the PATH bCPAP kit with oxygen blenders by healthcare workers.	Scores of usability by healthcare workers about ease of device set-up, learning the device, difficulty of use satisfaction with device and desire for continued use.	• HCW usability form	We will use frequency t[illegible] to summarize the responses on the Likert scale.<br><br>A total score generated summing 10 items. Means, standard deviations and ranges [illegible] be reported.
Objective 2: To assess <u>acceptability</u> of the PATH bCPAP kit with oxygen blenders by healthcare worker.	Themes from HCW interview questions about what is liked best, least about the PATH bCPAP and blenders, ease-of-use initially and over time, difficulty of use, adequacy of blends available, adequacy of training, what could be changed, would HCW continue to use the PATH kit with or without blenders if they were available after the study end?	• HCW Initial Interview Guide<br>• HCW Follow-up Interview Guide	Notes from in-depth interviews will transcrib[illegible] and imported in NVivo software, analysed usin[illegible] content and thematic techniques (mainly inductive approach)
Secondary objective: To report clinical characteristics, demographics and outcomes of patients treated with the PATH bCPAP kit and blenders in a newborn care unit in rural Uganda	Demographics and clinical characteristics	• Patient Data Form 1 (Demographics and Admission Clinical Data)	Frequencies, proportio[illegible] and totals will be used [illegible] summarize categorical data with median and interquartile ranges for continuous variables.

Makerere University School of Public Health • Higher Degrees, Research and Ethics Committee •
Valid Thru:
04 MAY 2022

5.9 Ethical considerations

5.9.1 Informed consent

Written, informed consent will be sought from the parents or legal guardian of every newborn started on bCPAP therapy, and who are available within 24 hours of initiation. When the legal guardians are available within 24 hours of bCPAP initiation, study personnel (nurses with experience in bCPAP therapy and blending, and fluent in Luganda) not caring for the infant will approach the legal guardians of eligible patients and inform them of the objectives of the research. Informed consent forms will be available in both English and Luganda and only IRB approved versions will be used. Study personnel obtaining consent will be fully trained on the recruitment and informed consent process and have human subjects research certification. The consent process is detailed in the Standard Operating Procedure for Obtaining Consent (Appendix E).

Patients:

Any patient who may benefit from care beyond that available at Kiwoko hospital are offered transfer (at their expense) to another facility. Meanwhile care for a newborn begins as soon as the patient is admitted. Using parental assent for admission and treatment is standard of care in the unit. Patients may require emergent initiation of bCPAP therapy due to respiratory failure. Patients often do not have a parent or legal guardian present at the time of bCPAP initiation, as the mother may still be in the operation theatre after a caesarean section, recovering from delivery, or away from the unit and unreachable by phone. In such instances, it is not appropriate to wait to obtain consent before the initiation of bCPAP therapy, as delaying the initiation of therapy could be life-threatening and would be contrary to WHO guidelines. (4)

If the patient does not have a parent of legal guardian available at the time of admission, standard of care will remain the same, and the patient will have bCPAP therapy initiated using the Kiwoko device as soon as it is clinically indicated. As soon as they are available to meet with study personnel, the parent or legal guardian will be approached for consent to the study and to the use of the PATH bCPAP kit and blender. Consent must be gained within 24 hours of bCPAP initiation to be eligible to participate in the study. If parents choose to consent, the Kiwoko bCPAP kit will be replaced with the PATH bCPAP kit and blender and the baby will be enrolled in the study. (Process described in section 5, study procedures).

If a parent or legal guardian is available at the time of admission, admitting staff will obtain an initial verbal consent from the parent to initiate them on the PATH bCPAP kit and blender. It may be inappropriate to pull the parents away for full consent at the time of admission due to: the time involved with obtaining the full consent process, the emergent nature of bCPAP therapy and the stress and pressure of having a sick newborn. In this instance, verbal consent will be gained. We will seek to obtain full written consent within 12 hours of receiving verbal permission. Please see the stand-alone verbal consent script, which is also included in the Consent Standard Operating Procedure (Appendix E). If after the full consent process, the parents no longer want to be a part of the study, the patient will be changed back to standard of care for the unit. The parents will receive a clear explanation of the alternative, including whether an air compressor is available for blending. If an air

Makerere University School of Public Health • Higher Degrees, Research and Ethics Committee
Valid Thru: 04 MAY 2022

compressor is not available at that time, we will state clearly that their baby may receive 100% oxygen and the potential consequences of such. Study information that has been gathered up to this point will not be retained by study personnel.

Formal consent process

Study personnel (nurses with experience in bCPAP therapy and blending, and fluent in Luganda) not caring for the infant will inform parent or legal guardian of the objectives of the research and obtain consent. This consent will be obtained in a private location within the newborn unit as available. If the parent or legal guardian is unable to read, an impartial witness will be present to ensure they understand the study and their rights. Risks as well as benefits and alternatives to study participation will be discussed. The parent or legal guardian will be informed that if they decline study participation, the patient will be treated with Kiwoko bCPAP kit, and oxygen will be blended if an air compressor is available. Study personnel will ask for parents to explain back to them the study intervention. Consent forms will include a contact of the Makerere IRB chairperson. Consent will be documented with parental signature or thumbprint.

Parents will have access to in-unit study personnel 24x7 to ask further questions (in Luganda or English). The parent or legal guardian can withdraw from the study at any time during the study.

Determination of continued device use will be at the discretion of the attending medical provider.

Healthcare worker consent:

Healthcare workers (nurses and midwives) who care for patients receiving bCPAP therapy with or without blending in the Kiwoko unit will be approached to participate in the study by research personnel who are not in a supervisory position to the worker.

Risks as well as benefits and alternatives to study participation will be discussed. Consent forms will include a contact of the Makerere IRB chairperson. Consent will be documented with signature. Consent will be obtained for obtaining photographs, video or audio recording of the healthcare worker or they may decline this.

Healthcare personnel will have access to the country principal investigator (PI) to ask further questions. The health care worker can withdraw from the study at any time during or after the study is complete.

5.9.2 Confidentiality

All subjects' confidentiality will be protected by not including names in data forms nor any personal identifiers in study report. Each participant will be assigned a study number and this linkage list/key will be kept separately from study forms, stored in a locked cabinet in a hospital office. The study code that links the study number to each participant will be destroyed immediately upon determination that the data set is final and locked.

Makerere University School of Public Health • Higher Degrees, Research and Ethics Committee • Valid Thru: 04 MAY 2022

- Study subjects (nor their parents/guardians) will not be contacted after the study for any reason.
- Paper data collection forms will be accessible only to the data collectors, data entry team, medical monitor and principal investigators. They will be stored in a locked cabinet in a hospital office. They will be maintained indefinitely after the completion of the study by Adara Development as long as secure storage is available.
- Video or audio recording of patients and/or healthcare worker observations and interviews will be maintained on the recording device which will be stored in a locked cabinet in a hospital office. The files will be uploaded to a password protected Adara server.
- Database software (REDCap [38]) will be maintained on the Adara server and password protected—only accessible by the data entry personnel, PIs, database designer, and biostatisticians/ data analysis team. These de-identified electronic databases may be maintained after the study time period per usual practices in password-protected database based at Adara.
- Funding recipients of Grand Challenges Canada (the sponsor of this study) must deposit data arising from Grand Challenges Canada-funded research into open access repository. All data in the repository will not include any personal identifiable information.

5.9.3 Risks, risk mitigation and adverse event reporting

Participation in this study does not raise the underlying risk associated with neonatal respiratory failure necessitating bCPAP therapy (i.e., death, disability, lung disease) in a resource-constrained setting. The rate of complications of the PATH bCPAP kit with blender should not differ from other improvised bCPAP kits as recommended by WHO. The baseline mortality for patients treated with bCPAP therapy in the Kiwoko Unit is 34%[35]. Most complications of bCPAP therapy are mild and can be addressed through careful bedside nursing management.[39]

Mild Physical Risks:

- Nasal irritation: A bCPAP system uses a nasal cannula as the patient interface. It is important that the cannula have a close fit in the nasal openings to prevent any loss of pressure. Multiple sizes of cannula are provided in the PATH kit to allow proper sizing for each patient. There is a risk of skin irritation from the nasal cannula due to rubbing or pressure from the cannula on surrounding areas. Standard of care for a patient on bCPAP therapy includes skin assessments every 3 hours. Proper sizing and positioning of the cannula, along with diligent skin care can prevent this complication.
- Abdominal distention: The bCPAP system works by administering a gentle flow of pressure into the infant's lungs to help them breath. An unavoidable but mild complication results from the flow of pressure that also enters the oesophagus and into the stomach. Accumulation of air in the stomach can lead to mild distention of the abdomen. An orogastric tube inserted into the patient's stomach and attached to an empty syringe (no plunger) will help eliminate any accumulated air. Positioning the patient to rest in a prone position can also help with abdominal distention.

Makerere University School of Public Health • Higher Degrees, Research and Ethics Committee • Valid Thru: 04 MAY 2022

Moderate Physical Risks:

- Nasal skin breakdown: With extended use, irritated skin surrounding the nasal openings may breakdown. Care includes diligent skin care and close observation. As tolerated, short breaks may be used to allow the skin time to heal. Discontinuation of bCPAP therapy may be an option if the infant's condition allows. This complication can be avoided with frequent assessment, proper sizing and positioning of the cannula, along with diligent skin care.
- Feeding intolerance: Accumulation of air in the stomach and intestines can lead to feeding intolerance (decreased digestion of milk, vomiting). Ensuring proper placement of the orogastric tube will help decrease this complication. The patient should receive a thorough examination to rule out other abdominal pathology that might be causing the distention. Smaller, more frequent feedings and placing the prone can also help to improve tolerance of feedings.

Severe Physical Risks:

- Hypoxia/hyperoxia: All use of oxygen poses a risk of hyperoxia when the amount delivered is higher than required. Blending oxygen with air provides the ability to administer levels of oxygen less than 100%. This poses a risk of hypoxia, when the percentage of oxygen administered is less than the patient requires. However blended oxygen is the only safe way to administer oxygen per WHO guidelines.[7] Risk of both hyperoxia and hypoxia is reduced by closely monitoring the patient's oxygen saturations via pulse oximetry. All patients enrolled in the study will receive continuous monitoring by pulse oximetry.
- Pneumothorax: Pneumothorax (reported rate of pneumothorax with bCPAP therapy in high resource settings in the literatures is 1.9%).[39] This can be life-threatening, but often can be treated with needle decompression, as is the standard of care at Kiwoko.[39] Early identification and prompt intervention are important to decrease risks of this complication. Refresher training will include assessment, identification and treatment for pneumothorax.

There is no psychological, social nor economic risk to the patient nor family with study participation when compared to standard of care in the unit.

There is no psychological, social nor economic risk to healthcare workers that participate in this study.

Risk Mitigation

The Kiwoko Hospital newborn care unit has been providing bCPAP therapy since 2012. The nursing staff are knowledgeable and experienced in the care of the patient receiving bCPAP therapy. Additional refresher training will take place before the study to ensure standardization of care. Patients will be closely monitored around the clock for complications of bCPAP use. Given need to titrate oxygen to saturations, patients will be monitored via continuous pulse oximetry.

Makerere University School of Public Health • Higher Degrees, Research and Ethics Committee • Valid Thru: 04 MAY 2022

Table 14: Adverse Events and Mitigation of Risk

Event	Mitigation

Mild adverse events:	
Nasal irritation	• Provide diligent skin care<br>• Ensure proper fit, size, and position of cannula, add padding when indicated
Abdominal distention	• Ensure orogastric tube is allowing accumulated air in the stomach to be vented between feeds<br>• Position infant prone
• **Moderate adverse events:**	
Nasal breakdown	• Provide diligent skin care<br>• Ensure proper fit, size, and position of cannula, add padding when indicated<br>• Consider short time periods off bCPAP if tolerated or discontinue bCPAP therapy if appropriate
Feeding intolerance	• Exam/review to screen for abdominal pathology<br>• Ensure orogastric tube is allowing accumulated air in the stomach to be vented between feeds<br>• Consider adjusting feeding volume or timing<br>• Position infant prone
• **Serious adverse events:**	
Hypoxia/Hyperoxia	• Continuous monitoring with pulse oximetry<br>• Adjustment of oxygen concentration according to patient need
Pneumothorax	• Early identification (diagnosed by sudden deterioration, unilateral loss of breath sounds, bedside X-ray or ultrasound as available)<br>• Needle thoracentesis
Death (34% expected mortality rate on bCPAP at Kiwoko)	

Care for injury

If a baby has an injury or illness from the study device or the procedures required for this study, the reasonable medical expenses required to treat such injury or illness will be paid for by Adara, Kiwoko, PATH or another relevant party who is involved in conducting the investigation (collectively referred to as the Study Collaborator/s). Free medical treatment will be provided at Kiwoko Hospital for any trial-related injuries, as determined by the Primary Investigator and Study Collaborators of the Study. If necessary, the costs of referral and management of an illness will be also be paid for.

The coverage for such injury or illness is only available if the principal investigator and the Study Collaborators, where relevant, have decided that the injury/illness is directly related to the device or procedures and is not the result of a pre-existing condition or the normal progression of the baby's medical condition. In particular, the independent medical monitor

Makerere University School of Public Health
Valid Thru:
04 MAY 2022
Higher Degrees, Research and Ethics Committee

Mild adverse events:	
Nasal irritation	• Provide diligent skin care<br>• Ensure proper fit, size, and position of cannula, add padding when indicated
Abdominal distention	• Ensure orogastric tube is allowing accumulated air in the stomach to be vented between feeds<br>• Position infant prone
• **Moderate adverse events:**	
Nasal breakdown	• Provide diligent skin care<br>• Ensure proper fit, size, and position of cannula, add padding when indicated<br>• Consider short time periods off bCPAP if tolerated or discontinue bCPAP therapy if appropriate
Feeding intolerance	• Exam/review to screen for abdominal pathology<br>• Ensure orogastric tube is allowing accumulated air in the stomach to be vented between feeds<br>• Consider adjusting feeding volume or timing<br>• Position infant prone
• **Serious adverse events:**	
Hypoxia/Hyperoxia	• Continuous monitoring with pulse oximetry<br>• Adjustment of oxygen concentration according to patient need
Pneumothorax	• Early identification (diagnosed by sudden deterioration, unilateral loss of breath sounds, bedside X-ray or ultrasound as available)<br>• Needle thoracentesis
Death (34% expected mortality rate on bCPAP at Kiwoko)	

Care for injury

If a baby has an injury or illness from the study device or the procedures required for this study, the reasonable medical expenses required to treat such injury or illness will be paid for by Adara, Kiwoko, PATH or another relevant party who is involved in conducting the investigation (collectively referred to as the Study Collaborator/s). Free medical treatment will be provided at Kiwoko Hospital for any trial-related injuries, as determined by the Primary Investigator and Study Collaborators of the Study. If necessary, the costs of referral and management of an illness will be also be paid for.

The coverage for such injury or illness is only available if the principal investigator and the Study Collaborators, where relevant, have decided that the injury/illness is directly related to the device or procedures and is not the result of a pre-existing condition or the normal progression of the baby's medical condition. In particular, the independent medical monitor

Makerere University School of Public Health • Higher Degrees, Research and Ethics Committee • Valid Thru: 04 MAY 2022

will be involved in the determination of whether an injury in a study participant is related to study procedures or device (and not the underlying infant's health condition).

Adverse Event Reporting

The standard of care will be followed as with any complications that results from/are associated with bCPAP use. Complications can be reported on the "HCW Observation of bCPAP Set-Up With or Without Blenders" form or the "Patient Data Form 2—Clinical Course and bCPAP Settings" and then will be reported on the adverse event record as described below.

Adverse events will be recorded on Adverse Event Record (Appendix D) and be treated as follows:

- **Mild Adverse Events:** assessed and followed by study personnel, treated by nurses and closely monitored until resolved.

- **Moderate adverse events:** assessed and addressed by country PI, monitored closely until resolution, recorded and reported to the IRB (as required) as well as notifying medical monitor within 72 hours.

- **Serious adverse events:** assessed and addressed by country PI, monitored closely until resolution, recorded and reported to the IRB (as required) as well as notifying medical monitor within 24 hours.

In addition, Unanticipated Adverse Device Effects (UADE) will be monitored and reported using the UADE Event Record (Appendix D). The USFDA defines a UADE as: "any serious adverse effect on health or safety or any life-threatening problem or death caused by, or associated with, a device, if that effect, problem, or death was not previously identified in nature, severity, or degree of incidence in the investigational plan or application (including a supplementary plan or application), or any other unanticipated serious problem associated with a device that relates to the rights, safety, or welfare of subjects".

Managing and reporting unanticipated problems or protocol deviations

Unanticipated problems:

- Any breaches in confidentiality will be remedied immediately by the study team and reported to the country PI.
- Protocol deviations will be addressed by the country PI (to ensure the best care of the patient) and protocol deviations will be noted in the records for that patient.
- Changes at the site that affect the conduct of the research will be addressed by the study PI and, as necessary, reported to the Makerere IRB and PATH's Research Ethics Committee.

5.9.4 Study and safety monitoring

Safety of the participants is of utmost importance. Patients will have continuous pulse oximetry monitoring and frequent physical and vital sign assessment. The average nurse to patient ratio in the newborn care unit is 1:4. Patients are reviewed daily by a doctor and a

Makerere University School of Public Health • Higher Degrees, Research and Ethics Committee • Valid Thru: 04 MAY 2022

doctor is on call 24x7 for the newborn care unit. The country PI will be available for consultation and an independent medical monitor will review moderate and severe adverse events (as described below). Patients requiring higher level of care (need for mechanical ventilation) will be offered transfer to a referral centre at their own expense, as is standard of care in unit.

This study will have an independent medical monitor to evaluate adverse events and make recommendations for continuing or stopping a trial instead of a Data Safety Monitoring Board (DSMB) for the following reasons:

1. the local investigators will have access to all data and
2. patients will be accrued too quickly to allow for a DSMB to complete safety monitoring.

The medical monitor is a Ugandan physician experienced in the care of neonates and use of bCPAP. They will be available via mobile phone or computer to review moderate and severe adverse events within 48 hours and discus findings with the principal investigator.
Study Stop Criteria: If the following criteria are met the medical monitor will report to the IRBs, study sponsor and may stop the study:

1. Mortality rises to 50% of patients treated with bCPAP therapy, assessed over 10 patients. Baseline mortality of patients on bCPAP therapy at Kiwoko (recorded from 2013- 2018) is 34% (standard deviation 8%)).[35] This threshold would be triggered by the death of 5 patients out of 10 which is 2 standard deviations above the baseline (34% + 16%).
2. Pneumothoraces are detected in 15% or more of patients treated with bCPAP therapy, assessed over 10 patients (1.9% occurrence reported in literature).[39]

In addition, the study will be stopped if a UADE results in patient injury that is unresolved or requiring medical intervention to resolve.

5.9.5 Benefits

Individuals treated with bCPAP therapy (whether in the study or not) will have the benefit of closer monitoring as they will be on continuous pulse oximetry. (The current standard of care for the unit allows for intermittent pulse oximetry once the patient is stable).

The research assistants will also check the set-up of the bCPAP circuit and can alert nurse if set-up incorrectly or if they have any concerns about patient safety.

All study patients also will all have access to blended oxygen to decrease their risk of oxygen toxicity. (The current standard of care for the unit is blending oxygen only when air compressors are functioning. There are times where there are not enough functioning air compressors, which leaves patients without blended oxygen.)

Once testing is completed and product revision finalized, the PATH kit and blenders have the potential to improve quality of care for newborns in Uganda and beyond. It will allow resource-constrained settings to provide bCPAP therapy while reducing the risk of morbidity

Makerere University School of Public Health • Higher Degrees, Research and Ethics Committee • Valid Thru: 01 MAY 2022

from oxygen toxicity. This low-cost device has the potential to prevent and estimated 178,000 neonatal deaths in Africa each year, and many more across the globe.[27]

Healthcare workers who participate in this study will benefit from receiving refresher training on diagnosis and management of respiratory distress syndrome in newborns.

Participants and their families will not receive compensation for participation in this study. Healthcare workers will not receive compensation for participation in this study.
~~Staff at Kiwoko Hospital will also benefit from exposure to research studies, giving them an opportunity to build their skills in this area.~~

Makerere University School of Public Health • Higher Degrees, Research and Ethics Committee •
Valid Thru:
04 MAY 2022

References

1 Silverman WA, Andersen DH: A controlled clinical trial of effects of water mist on obstructive respiratory signs, death rate and necropsy findings among premature infants. Pediatrics 1956;17:1-10.

2 Institute for Health Metrics and Evaluation: Global Burden Disease Compare Data Visualization. Seattle, WA, Institute for Health Metrics and Evaluation. University of Washington, 2017, 2017,

3 Schindler T, Koller-Smith L, Lui K, Bajuk B, Bolisetty S, New South W, Australian Capital Territory Neonatal Intensive Care Units' Data C: Causes of death in very preterm infants cared for in neonatal intensive care units: a population-based retrospective cohort study. BMC Pediatr 2017;17:59.

4 World Health Organization: WHO recommendations on newborn health: guidelines approved by the WHO Guidelines Review Committee; in Organization WH (ed). Geneva, WHO, 2017 (WHO/MCA/17.07),

5 McCollum ED, Smith AG, Eckerle M, Mvalo T, O'Brien KL, Baqui AH: CPAP treatment for children with pneumonia in low-resource settings. Lancet Respir Med 2017;5:924-925.

6 Tagare A, Kadam S, Vaidya U, Pandit A, Patole S: Bubble CPAP versus ventilator CPAP in preterm neonates with early onset respiratory distress--a randomized controlled trial. Journal of tropical pediatrics 2013;59:113-119.

7 World Health Organization: Oxygen Therapy for Children: A Manual for Health Workers. Geneva, Switzerland, 2016,

8 Chen A, Deshmukh AA, Richards-Kortum R, Molyneux E, Kawaza K, Cantor SB: Cost-effectiveness analysis of a low-cost bubble CPAP device in providing ventilatory support for neonates in Malawi inverted question mark a preliminary report. BMC Pediatr 2014;14:288.

9 Litch J, Robb-McCord J, Kak L: Safe and Effective Oxygen Use for Inpatient Care of Newborns; in Every Preemie- SCALE, USAID (eds): Do No Harm Technical Brief, Every Preemie- SCALE, USAID, 2017,

10 Oxygen Therapy for Children: A Manual for Health Workers; in Organization WH (ed). Geneva, Switzerland, World Health Organization, 2016,

11 United Nations Inter-agency Group for Child Mortality Estimation (UN IGME): Levels & Trends in Child Mortality: Report 2019; in Fund UNCs (ed). New York, 2019,

12 WHO: Born Too Soon: The Global Action Report on Preterm Birth.; in March of Dimes PfMNCH, Save the Children, World Health Organization (WHO). (ed). Geneva, March of Dimes, Partnership for Maternal Newborn Child Health, Save the Children, World Health Organization (WHO), 2013,

13 Uganda Demographic and Health Survey 2016 in Statistics UBo (ed). Kampala, Uganda, Uganda Bureau of Statistics, 2018,

14 World Health Organization: Survive and Thrive: Transforming Care for Every Small and Sick Newborn. Geneva, World Health Organization, 2019,

15 Carns J, Kawaza K, Liaghati-Mobarhan S, Asibon A, Quinn MK, Chalira A, Lufesi N, Molyneux E, Oden M, Richards-Kortum R: Neonatal CPAP for Respiratory Distress Across Malawi and Mortality. Pediatrics 2019;144

16 Barros FC, Bhutta ZA, Batra M, Hansen TN, Victora CG, Rubens CE, Group GR: Global report on preterm birth and stillbirth (3 of 7): evidence for effectiveness of interventions. BMC pregnancy and childbirth 2010;10 Suppl 1:S3.

17 Ho JJ, Henderson-Smart DJ, Davis PG: Early versus delayed initiation of continuous distending pressure for respiratory distress syndrome in preterm infants. The Cochrane database of systematic reviews 2002:CD002975.

18 Bharadwaj SK, Alonazi A, Banfield L, Dutta S, Mukerji A: Bubble versus other continuous positive airway pressure forms: a systematic review and meta-analysis. Archives of disease in childhood Fetal and neonatal edition 2020

Makerere University School of Public Health
Higher Degrees, Research and Ethics Committee
Valid Thru:
04 MAY 2022

19 Welty S: Continuous Positive Airway Pressure Strategies with Bubble Nasal Continuous Positive Airway Pressure. Clinics in perinatology 2016;43:661-671.
20 Norgaard M, Stagstrup C, Lund S, Poulsen A: To Bubble or Not? A Systematic Review of Bubble Continuous Positive Airway Pressure in Children in Low- and Middle-Income Countries. Journal of tropical pediatrics 2019
21 Carns J, Kawaza K, Liaghati-Mobarhan S, Asibon A, Quinn MK, Chalira A, Lufesi N, Molyneux E, Oden M, Richards-Kortum R: Neonatal CPAP for Respiratory Distress Across Malawi and Mortality. Pediatrics 2019
22 Thukral A, Sankar MJ, Chandrasekaran A, Agarwal R, Paul VK: Efficacy and safety of CPAP in low- and middle-income countries. Journal of perinatology : official journal of the California Perinatal Association 2016;36 Suppl 1:S21-28.
23 Okello F, Egiru E, Ikiror J, Acom L, Loe K, Olupot-Olupot P, Burgoine K: Reducing preterm mortality in eastern Uganda: the impact of introducing low-cost bubble CPAP on neonates <1500 g. BMC pediatrics 2019;19:311.
24 McAdams RM, Hedstrom AB, DiBlasi RM, Mant JE, Nyonyintono J, Otai CD, Lester DA, Batra M: Implementation of Bubble CPAP in a Rural Ugandan Neonatal ICU. Respir Care 2015;60:437-445.
25 World Health Organization: WHO recommendations for prevention and treatment of pre-eclampsia and eclampsia; in Organization WH (ed). Geneva, World Health Organization, 2011,
26 Diamedica Baby CPAP,
27 Brown J, Machen H, Kawaza K, Mwanza Z, Iniguez S, Lang H, Gest A, Kennedy N, Miros R, Richards-Kortum R, Molyneux E, Oden M: A high-value, low-cost bubble continuous positive airway pressure system for low-resource settings: technical assessment and initial case reports. PLoS ONE 2013;8:e53622.
28 WHO: WHO Model List of Essential Medicines; in WHO (ed). Geneva, WHO, 2017,
29 Blencowe H, Lawn J, Vazquez T, Fielder A, Gilbert C: Preterm-associated visual impairment and estimates of retinopathy of prematurity at regional and global levels for 2010. Pediatr Res 2013;74:35-49.
30 Adio A, Ugwu R, Nwokocha C, Eneh A: Retinopathy of prematurity in port harcourt, Nigeria. ISRN Ophthalmol 2014;481527
31 Mayet I, Cockinos C: Retinopathy of prematurity in South Africans at a tertiary hospital: A prospective study. Eye (Lond) 2006;20:29-31.
32 Van der Merwe S, Freeman N, Bekker A, Harvey J, Smith J: Prevalence of and risk factors for retinopathy of prematurity in a cohort of preterm infants treated exclusively with non-invasive ventilation in the first week after birth. 2013;103:96-100.
33 World Health Organization: WHO Recommendations on Interventions to Improve Preterm Birth Outcomes. Geneva, Switzerland, 2015,
34 US Department of Health and Human Services Food and Drug Administration: Information Sheet Guidance For IRBs, Clinical Investigators, and Sponsors; in US Department of Health and Human Services Food and Drug Administration (ed), 2006, 2017,
35 Hedstrom A, Lester D, Otai C, Nyonyintono J, Batra M: Kiwoko Hospital Neonatal Intensive Care Unit Database, 2019,
36 Hedstrom A, Ryman T, Otai C, Nyonyintono J, McAdams RM, Lester D, Batra M: Demographics, clinical characteristics and neonatal outcomes in a rural Ugandan NICU. BMC pregnancy and childbirth 2014;14:327.
37 Hedstrom A, Ryman T, Otai C, Nyonyintono J, McAdams R, Lester D, DiBlasi R, Batra B: Bubble CPAP Utilization and Outcomes in a Rural Ugandan NICU. PAS/ASPR Joint Meeting 2015
38 Harris PA, Taylor R, Thielke R, Payne J, Gonzalez N, Conde JG: Research electronic data capture (REDCap)--a metadata-driven methodology and workflow process for providing translational research informatics support. J Biomed Inform 2009;42:377-381.

Makerere University School of Public Health • Higher Degrees, Research and Ethics Committee
Valid Thru:
04 MAY 2022

39 Martin S, Duke T, Davis P: Efficacy and safety of bubble CPAP in neonatal care in low and middle income countries: a systematic review. Archives of disease in childhood Fetal and neonatal edition 2014;99:F495-504.

Makerere University School of Public Health • Higher Degrees, Research and Ethics Committee •
Valid Thru:
04 MAY 2022
