# Supplementary figures and images for: Feasibility and usability of a very low-cost bubble continuous positive airway pressure device including oxygen blenders in a Ugandan level two newborn unit
Source: PLOS Glob Public Health. 2023 Mar 8;3(3):e0001354. doi: 10.1371/journal.pgph.0001354 (PMC10021653; doi:10.1371/journal.pgph.0001354)

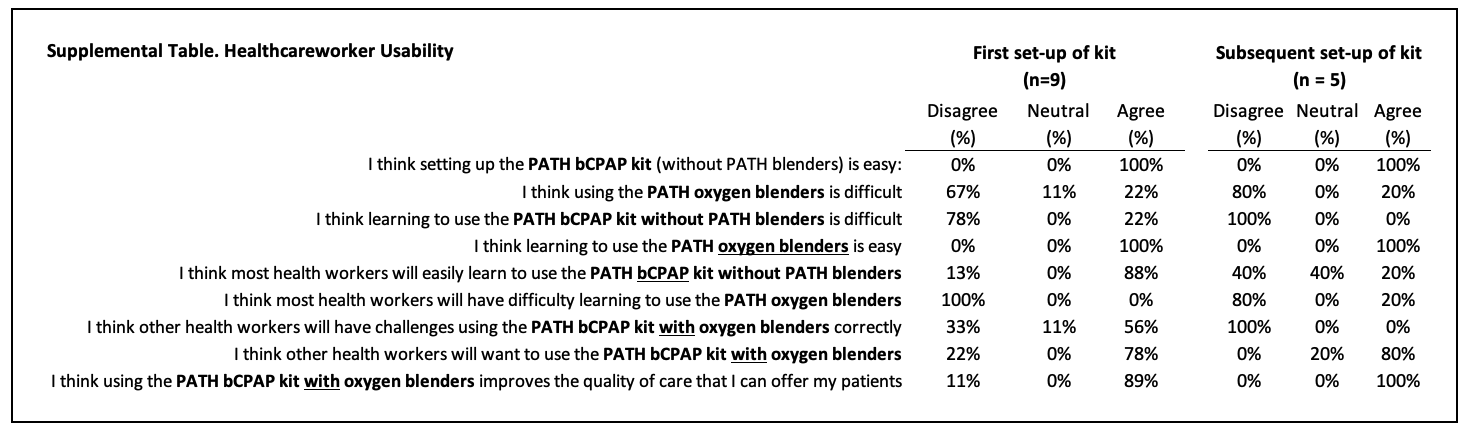

Supplement: S2 Table — (PNG) [file pgph.0001354.s009.png]
