# Supplementary material for: Feasibility and usability of a very low-cost bubble continuous positive airway pressure device including oxygen blenders in a Ugandan level two newborn unit
Source: PLOS Glob Public Health. 2023 Mar 8;3(3):e0001354. doi: 10.1371/journal.pgph.0001354 (PMC10021653; doi:10.1371/journal.pgph.0001354)
Supplement: Attachment — Submitted filename: Response to Reviewers.docx [file pgph.0001354.s010.docx]

Feasibility and Usability of a Very Low-Cost Bubble Continuous Positive Airway Pressure Device including Oxygen Blenders in a Ugandan Level Two Newborn Unit

Manuscript Number PGPH-D-22-01392R1

**Response to Reviewers**

Editor Comment:

We have received two detailed reviews from expert reviewers, both raising similar comments about the pre-clinical testing of this device (the entire bCPAP circuit, not just the blender) and requesting additional detail. Please provide **as much detail as you can on this testing** in supplemental material (especially if it has not been previously published in peer-reviewed literature) and **make clear in the manuscript itself what aspects have been tested, and what has not**.

Thank you for the opportunity to clarify the testing on the device. In an oversight, we did not reference the article on device testing (*Coffey PS. Nonclinical Bench Performance Testing of a Very Low-Cost Nonelectric Bubble Continuous Positive Airway Pressure (bCPAP) and Blenders Device Designed for Newborn Respiratory Support. Medical Devices: Evidence and Research. 2022*). It has now been cited and references to this testing added to the methods.

We appreciate that this article is focused on the feasibility and usability of this set-up - not on technical efficacy. But typically technical efficacy will have been demonstrated previously and this is not clearly communicated at present. If the device has not been fully tested or does not meet industry standards, please be transparent about what stage it is at with testing and validation. You may also wish to reflect on this in your discussion and conclusions, considering the assurances you would want to be able to use and recommend this set-up to others in the future (i.e. it has demonstrated feasibility and usability, but what else is important).

The reviewers also highlight some areas you can been **more measured in your conclusions**, clearer about comparison populations and reporting results, and provide sensible suggestions for improving readability and clarity to readers. I encourage you to take their suggestions on board as you revise.

Thank you for this suggestion, the methods have been edited (as described below).

Journal Requirements:

1. Please remove any photos of children (Figures 2) from your submission.

Black boxes have now been added over the baby’s eyes to figure 3 to protect patient privacy per journal submission guidelines. If necessary, the black boxes could be extended further over the face/body in the photos. However, this would impact the reader’s ability to see the clinical context of the patient care including placement of the Ram cannula in the nares. The parents provided written consent to the study for photos to be used in publication.

**Reviewer #1:**

Thanks for allowing me to review this important descriptive report of a Very Low-Cost Bubble Continuous Positive Airway Pressure Device including Oxygen Blenders in a Ugandan Level Two Newborn Unit. There is a good level of detail about the context and the process of evaluating the device. The strength is in the reporting of feasibility and acceptability among staff.

My main concern is that there is **inadequate data referenced or provided in the manuscript to feel confident the blender delivers what is intended when connected to a downstream high resistance system**. Please see corresponding comments under Results. There is a risk that policymakers/potential users take this report as validation of the effectiveness of the device, although the authors are rightly careful to not suggest that they aimed to evaluate effectiveness. As such, I suggest one or more of the following:

- **Explicitly state in the abstract and discussion that no evaluation was made of the effectiveness of achieving the specified FiO2 or PEEP**.

The abstract now states: “This study did not evaluate device effectiveness.”

The discussion now states: “...this was not a study of device effectiveness, and no evaluation was made of the effectiveness of achieving the specified FIO2 or PEEP.”

- Reference/provide as a supplement **further benchtop data** (? from Sinapi), or participant analysis as detailed in my comments below under Results.

Done (inserted references to Coffey 2022 benchtop testing data).

Abstract

Please **define PATH** before using the acronym

PATH is no longer an acronym and is now the legal name of the organization. (https://www.path.org/articles/what-does-path-stand-for/)

I note that that study was submitted to clinicaltrials.gov on June 16, 2022. The actual start date was October 4 2021 and completion date December 15, 2021. For transparency, the authors may want to **state in the abstract that this was retrospectively registered**.

Done, also in the methods

It is helpful that the protocol is submitted as a supplement.

Done (added S9 Fig)

The conclusion “may decrease risk of morbidity from oxygen toxicity” is not supported by the reported data. The authors may want to l**imit their conclusion to demonstration of feasibility and acceptability among staff**.

Done, the abstract conclusion has been changed to “... use of the bCPAP device including oxygen blenders is feasible and acceptable to healthcare workers.”

Methods.

Study setting. How is **thermoregulation** typically provided?

Added to text: “The unit provides thermoregulation via overhead warmers, incubators and kangaroo care”.

You state that “a **‘bubbler’ humidifier** located at the oxygen source” was used. However, this is not shown in Fig 1 or in the “bCPAP Device Characteristics” box. Please clarify.

Thank you for the chance to clarify. The passive oxygen bubbler was part of the oxygen source (compressor) used by the unit; it is not a part of the device.

The methods have been updated to “The device provides passive humidity provided by entrained ambient air. During the study additional humidity was provided by a ‘bubbler’ humidifier on the oxygen concentrator.”

The “bCPAP device characteristics box” now states “Humidity- Passive. The blender entrains room air with ambient humidity. In addition, the oxygen source (concentrator or tank/cylinder) may provide additional humidity via a bubbler bottle.”

Is there a **minimum recommended PSI for the oxygen source?** Particularly given the humidifier in the circuit?

Also, see point below re blender performance with downstream resistance. Blender performance may also be related to upstream pressure. Could the authors specify what oxygen source was used - cylinders vs concentrator and the implications for the use of the blender? If there is a minimum pressure requirement for the oxygen source for using the blender, you may want to specify this detail in the “bCPAP Device Characteristics” box.

The device was bench tested (Coffey 2022) over the range of pressures provided by medium and large oxygen concentrators (8.5 and 20 psi, respectively).

The methods text has now been updated to include:

- “A minimum of 8.5 psi oxygen source pressure is recommended for blender operation, which is achievable by many medium- or large-sized oxygen concentrators. *“*
- [in “bCPAP Device Characteristics” box]: “ Oxygen source requirements: 10LPM at 20psi for maximum blender performance, generally requires below 5LPM at 8.5 PSI for low birthweight patients”

Bubble humidifiers have a preset safety valve usually between 3-6 PSI that can limit the maximum flow capability of the blender (text has been updated and noted in response above). This lower pressure is able to provide sufficient flow for most preterm patients, but the limited blended flow rates can be inadequate for larger patients that require high flows. This was not an issue in the study which included term-sized infants.

The methods have been updated to “Use of the bubble humidifier limits the oxygen source pressure depending on safety valve setting (commonly 3-6 psi), which in turn reduces the maximum blended flow output capability of the blender.”

The **flow rates** used are reported in the Results section but how were these chosen for each participant? I see this is reported in the excellent CPAP guideline provided as a supplement but probably worth including in the main manuscript.

The methods now include “Flow rates were used to achieve gentle, continuous bubbling during the respiratory cycle without causing vigorous bubbling.”

If a higher oxygen flow rate is (perhaps accidentally) used, **does this affect the proportion of entrained air and therefore the delivered FiO2?**

Bench testing (Coffey 2022) showed the FIO2 was stable within 5% throughout the specified range of flows and pressures.

The results have been updated to include “[concentration of oxygen provided to the newborn] depends on the concentration of oxygen supplied to the blender (...) but remains within +/-5% of the blender’s nominal blend ratio for the full range of clinically relevant pressure (...) and despite changes in flows (...) for newborns.”

**Nasal foam pads-** please provide details of this and the source.

The methods text has now been updated to reflect these are “Neotech NeoSeal” pads that adhere to the nasal prongs. https://www.neotechproducts.com/product/neoseal/

How are the **RAM cannulae sized**- according the the manufacturer’s recommendation with a mandatory leak or do you size-up/use other strategies to minimise leak?

Yes the sizing of the RAM cannula allowed leak- the methods text now has been updated to include “The RAM cannula (Neotech) were sized to fill approximately 80% of the nares.” Of note the use of the nasal pad (described above) does reduce airleak.

Are the nurses trained to try and keep the mouth closed (perhaps with a strap), or is this not recommended at your site?

Response: Nurses are trained that, optimally, mouths are closed but no strap system is utilized.

Results.

In their bench top study, Mollazadeh-Moghaddam et al. (ref 31) state in the Discussion: “The primary limitation of our study is that **flow rates of mixed gas downstream of the blender were not measured**, and they may be affected by FiO2, upstream flow rates, oxygen loss through the entrainment window, and resistance of the downstream device… future studies are required to comprehensively evaluate more variables that impact blender performance, such as resistance and oxygen loss. The authors are currently optimizing an ambient air–oxygen blender for a variety of downstream devices (low/ high flow cannula, CPAP setups, etc.) with comprehensive measurements of these variables in separate studies. It is likely that unique blenders will need to be designed for individual downstream apparatus specifications.”

Consequently, there is **no data that the authors have referenced to feel confident about what flow rate and FiO2 is generated downstream of the blende**r/venturi device when connected to a high resistance circuit (RAM cannulae and under-water bubble CPAP). Are there other references/testing data available from Sinapi?

The now-referenced benchtop testing data (Coffey 2022) shows FIO_2_, pressure and flow rates downstream from the blender when connected to the Ram cannula as measured on a test lung. In addition, the manufacturer Sinapi verified the performance (flow, pressure, and FiO_2_) of every blender produced for the study.

Given the above, it seems important to understand (particularly in the absence of a control group) whether:

(1) **adequate pressure** was in fact achieved. Perhaps the authors could report the respiratory severity score not just before but also after bCPAP was commenced?

Downstream pressure exiting the RAM cannula was reported/confirmed as expected in Coffey 2022.

(2) the **downstream FiO2** was in fact at least close to what the researchers thought was being delivered. If downstream resistance is high, less room air may be entrained into the blender and delivered FiO2 may be higher than expected. This may have contributed to babies remaining hyperoxic despite the use of the 37% FiO2 blender. Did the researchers do any testing of the FiO2 exiting the RAM cannulae? If not, would the continuous SpO2 data shed some light here (perhaps a pre-post analysis of infants that were initially in 100% oxygen and then switched to the bCPAP device with blender)?

FIO2 exiting the RAM cannula was reported/confirmed as expected in Coffey 2022.

The authors report adverse events of episodes of hyperoxia, but **were there any adverse events of hypoxia**?

No hypoxia was reported as an adverse event, however the results have now been updated: “Adverse events included: episodes of hyperoxia (oxygen saturations >95% lasting greater than 5 minutes) in 14/14 patients and death *(including progressive hypoxia)* in 2/14 patients.”

Given the limited ability to titrate with the blenders, it would be good to **report percentage of time participants were within, below and above the target SpO2 range**.

Agreed this would be useful information. However, the study used manually recorded “spot checks” of oxygen saturation and did not have continuous recording of saturation. Therefore, we are unable to report the exact percent time above and below the target range.

Were there any instances of the **blender being attached in the reverse direction?**

One observation form for device set-up on a patient noted “no” to the question “blender is correctly placed within the inspiratory tubing of bCPAP circuit” but I do not have further information. This would have been noted by the observer and corrected so not a set-up error that reached the patient.

Discussion.

2nd paragraph: “Despite delivery of blended oxygen, each patient continued to have periods of **hyperoxia** (saturations >95%) as they had on nasal cannula oxygen before bCPAP initiation.” This sentence is confusing. Are the authors stating that the reported hyperoxia relates to when the infants were on low flow alone? Or did they also have hyperoxia while on blended bCPAP?

The text has been edited for clarity to: “Despite delivery of blended oxygen, each patient continued to have periods of hyperoxia (saturations >95%). This hyperoxia also occurred when these patients were on nasal cannula oxygen before bCPAP initiation.”

Please also discuss the limitations regarding delivered FiO2 not being evaluated, as mentioned above.

N/A

 Minor points

Background.

**Line 66**, “keep their lungs open” could be more scientifically phrased.

This has been edited to “maintain adequate lung expansion”.

Line 71-72, in what way is **bubble CPAP superior?** The reference cited relates to lower CPAP failure but higher nasal injury. Also, what is meant by “**standard**” CPAP?

Edited to “bCPAP is considered superior in treating neonatal respiratory failure compared with *flow-driven* CPAP because it results in *lower failure rates* and need for mechanical ventilation”

**Reviewer #2:**

Before being applied to patients this research should have had a **technical/benchtop peer reviewed engineering paper**. The background refers to references 25 and 26 but these are not peer reviewed publications (one a meeting abstract and the other a media piece). Do we even know that this bCPAP System provides flows, oxygen concentrations, pressures, resistance, and imposed work of breathing in ranges that are technically acceptable/reliable? What about **humidification**?

This is important feedback that has been addressed above. The benchtop testing paper (Coffey 2022) addresses humidification as well and some edits made on this topic as described in response to reviewer 1’s comments above.

With only 14 enrolled patients, a survey of 9 providers, with the confounder of a compressor that was used for priority patients, after use/implementation over 4 months in a referral facility newborn unit for 3 Districts (with a median census of 46) makes **statements on mortality** ("mortality rate below historical") **and scale** (".....the PATH bCPAP device may be ideal") an inappropriate stretch. Instead, this is a very early peek into a novel device which prompts a number of intriguing questions that inspire further study. It is far too early to suggest this is ready for use on patients given the information/data to-date.

This is important feedback, the discussion has been adjusted to:

“The mortality rate (14%) is difficult to interpret given the small sample size, but during the study was below the unit’s historical rates on bCPAP per unit records (34%). Overall, these outcomes are consistent with other settings lacking advanced respiratory therapies such as surfactant and mechanical ventilation.”

“For sites ready for bCPAP implementation where higher priced devices are out of reach, further study on the practicality and implementation of very low cost devices including the PATH bCPAP device may be ideal. Scale-up and access to more affordable bCPAP devices with the ability to blend oxygen are needed to achieve World Health Organization targets and improve facility-based care.*“*
